# Centromere Landscapes Resolved from Hundreds of Human Genomes

**DOI:** 10.1093/gpbjnl/qzae071

**Published:** 2024-10-18

**Authors:** Shenghan Gao, Yimeng Zhang, Stephen J Bush, Bo Wang, Xiaofei Yang, Kai Ye

**Affiliations:** School of Automation Science and Engineering, Faculty of Electronic and Information Engineering, Xi’an Jiaotong University, Xi’an 710049, China; School of Computer Science and Technology, Faculty of Electronic and Information Engineering, Xi’an Jiaotong University, Xi’an 710049, China; MOE Key Lab for Intelligent Networks & Networks Security, Faculty of Electronic and Information Engineering, Xi’an Jiaotong University, Xi’an 710049, China; School of Computer Science and Technology, Faculty of Electronic and Information Engineering, Xi’an Jiaotong University, Xi’an 710049, China; MOE Key Lab for Intelligent Networks & Networks Security, Faculty of Electronic and Information Engineering, Xi’an Jiaotong University, Xi’an 710049, China; School of Automation Science and Engineering, Faculty of Electronic and Information Engineering, Xi’an Jiaotong University, Xi’an 710049, China; School of Automation Science and Engineering, Faculty of Electronic and Information Engineering, Xi’an Jiaotong University, Xi’an 710049, China; MOE Key Lab for Intelligent Networks & Networks Security, Faculty of Electronic and Information Engineering, Xi’an Jiaotong University, Xi’an 710049, China; School of Computer Science and Technology, Faculty of Electronic and Information Engineering, Xi’an Jiaotong University, Xi’an 710049, China; MOE Key Lab for Intelligent Networks & Networks Security, Faculty of Electronic and Information Engineering, Xi’an Jiaotong University, Xi’an 710049, China; School of Automation Science and Engineering, Faculty of Electronic and Information Engineering, Xi’an Jiaotong University, Xi’an 710049, China; MOE Key Lab for Intelligent Networks & Networks Security, Faculty of Electronic and Information Engineering, Xi’an Jiaotong University, Xi’an 710049, China; Center for Mathematical Medical, The First Affiliated Hospital, Xi’an Jiaotong University, Xi’an 710061, China; School of Life Science and Technology, Xi’an Jiaotong University, Xi’an 710049, China; Faculty of Science, Leiden University, Leiden 2311 EZ, The Netherlands

**Keywords:** Centromere sequence, Higher order repeat annotation, Human population, High-fidelity sequencing technology, Genome evolution

## Abstract

High-fidelity (HiFi) sequencing has facilitated the assembly and analysis of the most repetitive region of the genome, the centromere. Nevertheless, our current understanding of human centromeres is based on a relatively small number of telomere-to-telomere assemblies, which have not yet captured its full diversity. In this study, we investigated the genomic diversity of human centromere higher order repeats (HORs) via both HiFi reads and haplotype-resolved assemblies from hundreds of samples drawn from ongoing pangenome-sequencing projects and reprocessed them via a novel HOR annotation pipeline, HiCAT-human. We used this wealth of data to provide a global survey of the centromeric HOR landscape; in particular, we found that 23 HORs presented significant copy number variability between populations. We detected three centromere genotypes with unbalanced population frequencies on chromosomes 5, 8, and 17. An inter-assembly comparison of HOR loci further revealed that while HOR array structures are diverse, they nevertheless tend to form a number of specific landscapes, each exhibiting different levels of HOR subunit expansion and possibly reflecting a cyclical evolutionary transition from homogeneous to nested structures and back.

## Introduction

Centromeres are essential yet rapidly evolving chromosomal domains with functional roles in cell division [1], although they are characteristically challenging to assemble [2]. Human centromere sequences typically comprise multiple alpha satellite monomers (∼ 171 bp in length and generally sharing 50%−90% identity) organized into higher order repeat (HOR) units (which share approximately 95%−100% identity) [3,4].

This high level of repetition ensures that centromeres are difficult to assemble and that reads cannot easily be mapped to them with high accuracy, collectively hindering investigations of centromere architecture and evolution [4,5]. However, advanced long-read sequencing technologies, particularly PacBio high-fidelity (HiFi) reads [6], have recently yielded several telomere-to-telomere (T2T) assemblies [2,7–9]. The T2T consortium published the first complete human genome (CHM13) in 2022 alongside an analysis of its centromeres [2,10]. This provided a detailed chromosome-specific HOR atlas and demonstrated that human centromeres evolved through a process of “layered expansion”, in which younger sequences expanded from the middle, resembling successive tandem duplications, with older flanking sequences shrinking and diverging over time [10]. A second T2T human genome has since been completed (CHM1) alongside analyses of both the genetic and epigenetic variations within its centromeres [9]. Yang et al. reported the new genetic and epigenetic variations of centromeres in a male Han Chinese genome [8].

Despite the substantial insight afforded by assembling genomes at the T2T level, in absolute terms, a small number of complete assemblies remain insufficient for characterizing the rapid evolution and diversity of centromere sequences. To that end, Suzuki et al. used long-read sequencing of 36 individuals to reveal the structural diversity of human centromere HORs [11]. However, their samples primarily comprised individuals from one population (Japanese) and their strategy was limited to characterizing the HOR patterns of chromosome 11 (Chr11), Chr17, and ChrX (collectively “suprachromosomal family 3”), as these were considered more divergent from other chromosomes [11,12]. Recently, both the Human Pangenome Reference Consortium (HPRC) and the Chinese Pangenome Consortium (CPC) released HiFi reads and haplotype-resolved assemblies of over one hundred individuals from a diverse range of ancestries, which, to the best of our knowledge, have not yet been used in a dedicated population-wide analysis of centromere sequence variation [13,14]. Combining data from these projects provides an opportunity to investigate the diversity and evolution of centromere sequences among the broader human population. Here, we refined and updated our previous HOR-annotation tool, HiCAT [15], which was initially designed only for use with an individual assembly, to annotate hundreds of centromeres. Using this improved version, HiCAT-human, we analyzed HiFi reads from 102 individuals and the assemblies of 109 haplotypes — collectively, every sample released by both the HPRC and CPC, plus CHM13, leveraging this wealth of data to provide a comprehensive global survey of the human centromeric landscape.

## Results

### HOR annotation in the human population

To investigate the diversity of human centromeres, we analyzed HPRC and CPC HiFi reads from 102 individuals including 22 from Africa (AFR, all from HPRC), 16 from Latin America (AMR, all from HPRC), 62 from East Asia (EAS, 4 from HPRC and 58 from CPC), and 1 from South Asia (SAS, from HPRC), plus CHM13. In addition, we analyzed 108 haplotype-resolved assemblies (43 × 2 from HPRC and 11 × 2 from CPC) alongside the CHM13 genome (Table S1) [2,13,14].

**Figure 1:**
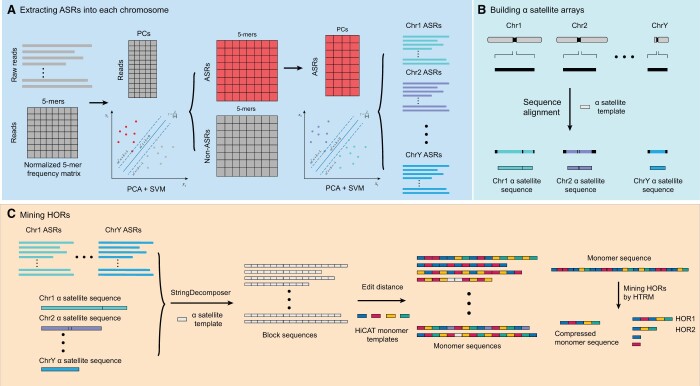
Overview of HiCAT-human **A**. Extracting reads containing alpha satellites and classifying them into each chromosome. The 5-mer frequency matrix constructed from raw reads (gray) is classified into ASRs and non-ASRs via PCA and SVM. Then, a 5-mer frequency matrix constructed from ASRs (red) is classified into each chromosome via PCA and SVM. **B**. Building alpha satellite arrays from the assembled genome. The black bars represent pericentromeric and centromeric regions. Regions inside bars with different colors represent alpha satellite regions in each chromosome. Sequence alignment is performed by Lastz [16] using the alpha satellite sequence as a template. **C**. Mining HORs. All chromosomes’ ASRs and satellite arrays are first transformed into block sequences via StringDecomposer [17] with the alpha satellite sequence as a template, and then the edit distance between the HiCAT monomer templates and blocks is used to transform block sequences into monomers. Finally, HORs are annotated via monomer sequences and the HTRM method [15]. Different colored rectangles in the monomer sequence represent different monomers. ASR, alpha satellite read; PCA, principal component analysis; PC, principal component; SVM, support vector machine; HOR, higher order repeat; HTRM, hierarchical tandem repeat mining.

We modified our HOR annotation tool HiCAT, which was originally designed to work with individual assemblies [15], to create an updated version, HiCAT-human, which can automatically annotate centromere HOR patterns from both reads and assemblies of multiple human samples (**Figure 1**, Figure S1; Tables S2 and S3; File S1). HiCAT-human comprises two workflows for this purpose, HiCAT-human-reads and HiCAT-human-assembly. In HiCAT-human-reads, alpha satellite reads (ASRs) are extracted and classified into each chromosome with a pretrained classifier (Figure 1A), and in HiCAT-human-assembly, alpha satellite regions in each chromosome are detected via Lastz [16] and then merged to obtain an alpha satellite array (Figure 1B). In the subsequent annotation step, both ASRs and satellite arrays were transformed into block sequences via StringDecomposer [17] and then into monomer sequences via monomer templates derived by HiCAT [15]. Monomer sequences were used to derive HORs following a hierarchical tandem repeat mining (HTRM) approach [15] (Figure 1C).

### Human HOR quantification on the basis of HiFi read data

**Figure 2:**
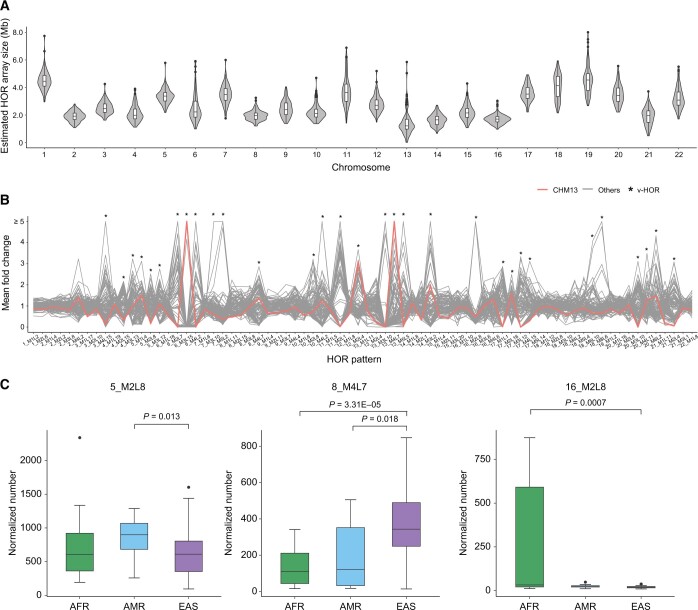
HOR annotation for the HiFi reads of 102 individuals **A**. Estimated HOR array size based on the total length of HOR reads and sequencing coverage on each autosome. **B**. The mean fold change in autosomal HORs among all samples. The mean fold change is calculated by dividing the n-number of HORs in each sample by the mean n-number of HORs across all samples. CHM13 is represented by a red line, and all other samples are represented by gray lines. v-HORs are marked by asterisks. The results for sex chromosomes are shown in Figure S2. **C**. The n-number of three representative HOR arrays (5_M2L8, 8_M4L7, and 16_M2L8) in the AFR (sample number = 22), AMR (sample number = 16), and EAS (sample number = 62) populations. Other v-HORs are shown in Figures S6 and S7. *P* value is calculated by two-sided Wilcoxon rank sum test. n-number, normalized number; v-HOR, variable HOR; AFR, Africa; AMR, Latin America; EAS, East Asia.

To identify human centromere HORs, we applied the HiCAT-human-reads workflow to the HiFi reads of 102 samples. We estimated the size of the HOR arrays for each chromosome in each sample as the total number of bases in the HOR reads divided by the sequencing coverage. The median estimated HOR array size varied from 0.8 to 4.5 Mb across all chromosomes (**Figure 2**A, Figure S2A) and showed marked variability between populations. We found that for eight chromosomes, the estimated HOR array sizes in the EAS populations were significantly larger than those in the AFR and AMR populations and that conversely, on Chr16, Chr21, and ChrY, the estimated array sizes were significantly larger in the AFR populations than in the other populations (Figures S3−S5; Table S4). To compare how the number of HORs varied among samples, we calculated the “n-number” as the total count of HORs in each sample normalized by the depth of sequencing coverage. For subsequent analysis, we excluded rare HORs to capture the main characteristics. In total, we obtained 79 HORs, 33 of which exhibited pronounced variance between populations (on the basis of a mean fold change in their n-number among samples), which were referred to as “variable HORs” (v-HORs) (Figure 2B; Tables S5−S12). This variation in normalized HOR counts demonstrates that the CHM13 genome represents only one distribution of the human HOR landscape and that, by extension, it is unable to capture the broader range of human HOR diversity (Figure 2B, Figure S2B and C). We found that 23 of the 33 v-HORs were significantly variable among populations (Figure 2C, Figures S6 and S7; Table S13), including 5_M2L8 (significantly higher in AMR than in EAS) and 8_M4L7 (significantly higher in EAS than in AMR and AFR).

### Chromosomal variability in centromere genotypes

**Figure 3:**
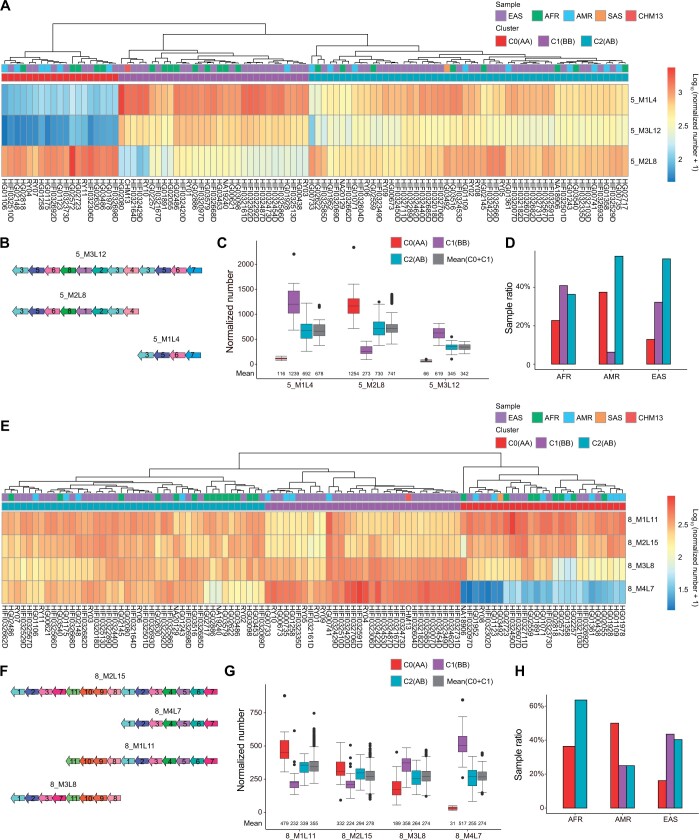
Centromere genotype from sample clustering based on HOR n-numbers on chromosomes 5 and 8 **A**. Heatmap and sample hierarchical clustering of HOR n-numbers on Chr5. **B**. Monomer patterns of 5_M3L12, 5_M2L8, and 5_M1L4. **C**. Box plot of HOR n-numbers in 5_C0(AA), 5_C1(BB), and 5_C2(AB). For each HOR, the mean(C0+C1) represents the pairwise mean n-numbers in 5_C0 and 5_C1. **D**. Proportion of samples in each of the AFR, AMR, and EAS populations containing 5_C0, 5_C1, and 5_C2. **E**. Heatmap and sample hierarchical clustering of HOR n-numbers on Chr8. **F**. Monomer patterns of 8_M2L15, 8_M1L11, 8_M3L8, and 8_M4L7. **G**. Box plot of HOR n-number among 8_C0(AA), 8_C1(BB), and 8_C2(AB). For each HOR, the mean(C0+C1) represents the pairwise mean n-numbers in 8_C0 and 8_C1. **H**. Proportion of samples in each of the AFR, AMR, and EAS populations containing 8_C0, 8_C1, and 8_C2.

To explore the distribution of HORs between chromosomes, we calculated the correlation between the n-number of different HORs in all samples and consistently found no significant correlation between inter-chromosomal HORs and a high correlation between intra-chromosomal HORs (Figure S8; Table S14), which suggests very little commonality between chromosomes in terms of their centromere composition. For each of the 17 chromosomes with v-HORs (*i.e.*, those chromosomes whose HORs show pronounced variation between samples), samples could generally be grouped into two or three clusters (**Figure 3**, Figures S9−S17; Table S15). Taking Chr5 as an example, we detected three clusters (termed 5_C0, 5_C1 and 5_C2), each with a variable composition of HORs (Figure 3A and B, Figure S9A). We found that the n-numbers of all three HORs in cluster 5_C2 approximated the average n-number of clusters 5_C0 and 5_C1 (Figure 3A and C; Table S16), which suggests that the latter are homozygous (AA and BB, respectively) and that by extension cluster 5_C2 is heterozygous (AB). Moreover, we found that 5_C2 was more frequently detected in each of the three main population groups (being present in 36%−56% of the samples), whereas 5_C0(AA) and 5_C1(BB) presented greater population bias (Figure 3D). Specifically, the EAS population had the significantly lowest proportion of samples with the 5_C0(AA) genotype (12.9%, *P* = 0.039 compared with AFR and *P* = 1.636E−05 compared with AMR; one-sided binomial test), whereas the AMR population had the significantly lowest proportion of 5_C1(BB) (6.3%, *P* = 0.003 compared with AFR and *P* = 0.017 compared with EAS; one-sided binomial test) (Figure 3D). We found similar results for three clusters of HORs on Chr8 (Figure 3E−G; Table S16) and Chr17 (Figure S17; Table S16). On Chr8, we found that no 8_C1(AA) was present in the AFR samples, and the sample ratios of 8_C0 in AMR (50.0%, *P* = 0.002) and AFR (36.4%, *P* = 0.017) were significantly higher than that in EAS (16.1%) (Figure 3H). On Chr17, three genotypes were reported in previous studies, with an allele frequency of B of 61.9% in the Japanese population [11] and 35% in the European population [18]. We found that the allele frequency of B in EAS is 47.6% (59 of 124), which is significantly higher than that in the European population (*P* = 0.0026) but lower than that in the Japanese population (*P* = 0.0008). Taken together, these results suggest that centromere genotypes are highly variable among and between populations.

### Chromosome-specific HOR landscapes

**Figure 4:**
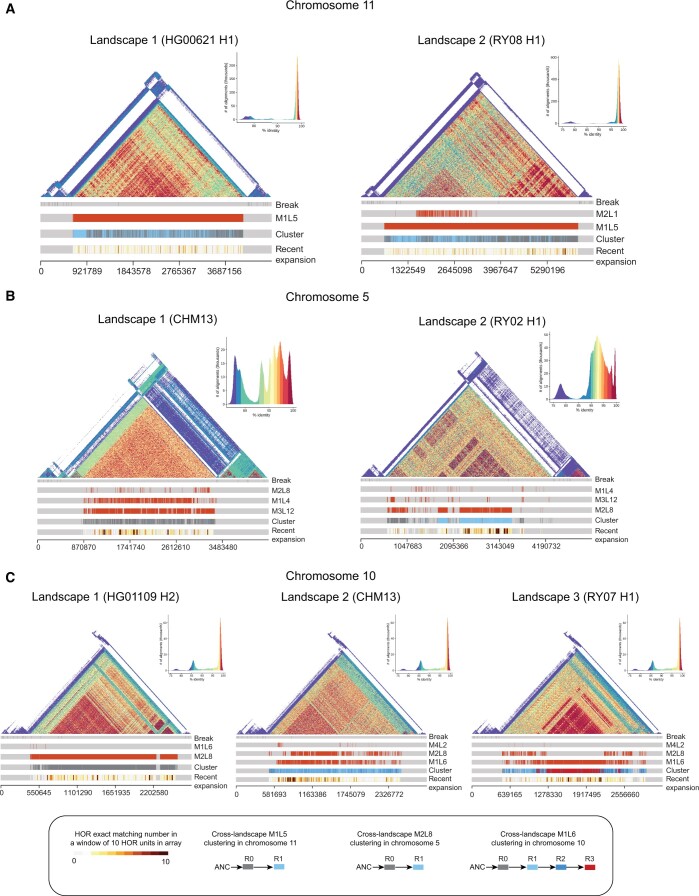
HOR landscapes on chromosomes 11, 5, and 10 The HOR landscapes on Chr11 represented by HG00621 haplotype 1 (H1) and RY08 H1 (**A**), on Chr5 represented by CHM13 and RY02 H1 (**B**), and on Chr10 represented by HG01109 H2, CHM13, and RY07 H1 (**C**). The triangle similarity heatmaps are generated by StainedGlass [31]. The “Break” track shows the breakpoints in satellite arrays. The “cluster” track represents the cross-landscape satellite array HOR clustering results. The “recent expansion” track represents the HOR exact matching number within a sliding window of 10 HOR units (with 1 HOR slide). The other tracks record the positions of different HORs. The ANC of each chromosome is reconstructed from monomers from other chromosomes with the same suprachromosomal family [12]. ANC, ancestral HOR sequence.

To develop our analysis beyond a population-level characterization of HOR diversity, we next investigated the distribution of HOR loci between samples. We applied HiCAT-human-assembly to 109 assemblies and annotated the HOR patterns in each satellite array. We then compared the distribution of HORs in satellite arrays between assemblies and found that the majority of chromosomes contained a discernible and different composition of HORs (hereafter “landscape”) and that 10 chromosomes seemingly contained two distinct landscapes (**Figure 4**A and B, Figures S18 and S19). For example, on Chr11, the first of the two landscapes is homogeneous, entirely consisting of one HOR (specifically, M1L5) with a repeating pattern of monomers of the form 1 − 2−3 − 4−5 (Figure 4A). In contrast, the second landscape demonstrates the expansion of a different HOR, M2L1 (monomer 1 local expansion), within a larger set of M1L5 arrays; this expansion arose from the tandem duplication of the first monomer in M1L5, which in this landscape has a repeating pattern of monomers of the form 1 − 1−2 − 3−4 − 5 (Figure 4A, Figure S20). This landscape of HORs has previously been detected in CHM1 [9]. We observed similar patterns for the satellite arrays of Chr3, Chr6, Chr12, Chr14, and Chr20 (Figures S18A, S18C, S18F, S19A, and S19E). The common feature of dual-landscape centromeres is that one landscape is homogeneous (dominated by one HOR), whereas the other landscape is “locally nested”, indicating the local expansion of a subunit within the primary HOR unit. We also found that the local expansion rates differed greatly between chromosomes, with relatively high rates for Chr11 but lower rates for Chr3 and Chr20 (Figure S21).

On Chr5, the variation in HOR composition is greater than that in the six aforementioned chromosomes (Figure 4B). The primary HOR in landscape 1 (sample number = 57) is M3L12, whereas that in landscape 2 (sample number = 52) is M2L8. Since M2L8 monomer pattern (MP) is a subunit of M3L12, we performed cross-landscape M2L8 clustering and found that cluster R0 in landscape 2 is shared with landscape 1, whereas cluster R1 is specifically enriched in landscape 2, constituting a new layer within it (Figure 4B, Figure S22).

We found three types of landscapes in the satellite array of Chr10: landscape 1, which primarily comprises HOR M2L8 (MP: 1 − 2−3 − 4−5 − 6−8 − 7) with a small number of M1L6 (MP: 1 − 2−3 − 4−5 − 6); landscape 2, which shows the co-occurrence of M2L8 and M1L6 across the entire satellite array; and landscape 3, which similarly shows the co-occurrence of M2L8 and M1L6 although with M1L6 expanding within the middle (Figure 4C). Cross-landscape M1L6 clustering revealed that cluster R0 is concentrated on landscape 1 and both ends of landscapes 2 and 3. R1 and R2 are interlaced in landscape 2 and both sides of landscape 3. R3 specifically exists in the middle of landscape 3 (Figure 4C, Figure S23). We compared the consensus sequences of three clusters with the reconstructed ancestral sequence and found that R0 is the most similar, whereas R3 may have more recently expanded (Table S17). On the basis of these results, we propose a model to illustrate the evolution of Chr10 HORs. The ancestral landscape may be homogeneous with M2L8 (landscape 1), with a deletion or local duplication event giving rise to M1L6, which expanded through the process of “layer expansion” [10] to form, initially, landscape 2, and then, after more extensive expansion, landscape 3.

We detected four types of landscapes of HORs on Chr8 (Figure S18E). We found that most samples (sample number = 62) belong to landscape 1, where M1L11 is concentrated on both sides of the satellite array, whereas M2L15 are enriched in the middle. Landscape 2 (sample number = 37) are represented by CHM13. Unlike landscape 1, M4L7 has a recent expansion in the middle of the satellite array. In addition to these two major landscapes, we also detected two minor landscapes: landscape 3, which was found in only 9 samples, with M1L11 concentrated to the right of the satellite array and M2L15 (with a locally nested M3L8) to the left; landscape 4, which is similar to landscape 1 with its center containing multiple copies of M3L8 and was found in only one sample (NA18906 H2).

Except for the abovementioned chromosomes, the other chromosomes contained only one HOR landscape among all the samples, and they were grouped into two types (Figures S24−S26). The first one includes Chr19, Chr22, and ChrX, and their satellite arrays are quite homogeneous dominated by a single HOR and similar among all samples. The second one contains many locally nested HORs (LN-HORs), such as Chr1, Chr2, Chr9, and Chr15. In summary, these results demonstrate that while human centromere HOR arrays are diverse, they share structural resemblances in their composition and thus form a number of “landscapes”.

### Different levels of LN-HORs contribute to the evolution of centromere landscapes

The abovementioned analysis suggests that LN-HOR plays a recurring role in the evolution of centromeres. The satellite array of Chr1 primarily comprises 1_M2L6 (MP: 1 − 2−5 − 6−4 − 3), with locally nested 1_M1L2 (a dimer, *i.e.*, with repeating pattern 1 − 2) (Figure S25A; Table S18). We found that the dimer expansion peak of 1_M2L6 is at the position of repeating four times with 12 monomers (12-mors) [(1 − 2)×4 − 5−6 − 4−3] [15]. We further analyzed the monomer length distribution of all the M2L6 units and found that there were different peak positions among all the samples (Figure S27). The peaks in 68.6% of the samples are at the position of three repeats with 10-mors [(1 − 2)×3 − 5−6 − 4−3], and the sample ratio with the peak at the position of 12-mors [(1 − 2)×4 − 5−6 − 4−3] is higher in EAS (32.3%) than in AFR (18.2%, *P* = 0.005, one-sided binomial test) or AMR (18.8%, *P* = 0.008, one-sided binomial test) (Figure S27G).

**Figure 5:**
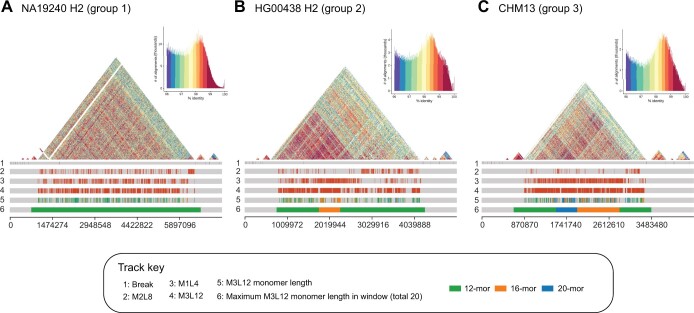
LN-HOR variation in chromosome 5 centromere arrays Chr5 satellite array landscapes represented by NA19240 H2 (**A**), HG00438 H2 (**B**), and CHM13 (**C**). The triangle similarity heatmaps are generated by StainedGlass [31], and those sequences with identities lower than 96% are excluded. The first track shows the breakpoints in satellite arrays. The second to fourth tracks show the positions of 5_M2L8, 5_M1L4, and 5_M3L12. The fifth track shows the positions of 12-mors (green), 16-mors (orange), and 20-mors (blue) of M3L12 units. In the sixth track, the entire satellite array is split into 20 windows with each window showing the maximum value of the total number of 12-mors, 16-mors, and 20-mors. 12-mor, 12 monomers; 16-mor, 16 monomers; 20-mor, 20 monomers.

Our previous study on CHM13 centromere HORs revealed that the 5_M3L12 units in the Chr5 centromere have three frequent MPs with 12-mors [3 − 5−6 − 8−1 − 2−3 − 4−(3 − 5−6 − 7)×1], 16-mors [3 − 5−6 − 8−1 − 2−3 − 4−(3 − 5−6 − 7)×2], and 20-mors [3 − 5−6 −8−1 − 2−3 − 4−(3 − 5−6 − 7)×3] [15]. We wondered whether these three MPs had different frequencies among samples with different patterns. The satellite array of CHM13 Chr5 belongs to landscape 1, where 5_M3L12 exists with M1L4 expansion in the entire array. We analyzed on landscape 1 and revealed that the ratio distributions of these three MPs from M3L12 can be clustered into three groups. In group 1 (27 samples), the ratio of 12-mors is high, and those of 16-mors and 20-mors are low, whereas in group 2 (18 samples), the ratio of 16-mors is high relative to group 1. In group 3 (12 samples), which includes CHM13, all three MPs are present at high frequencies (Figure S28; Table S19). Taking NA19240 H2 (group 1), HG00438 H2 (group 2), and CHM13 (group 3) as examples, the entire satellite array of NA19240 H2 is represented by a 12-mor MP, whereas the middle region of the HG00438 H2 satellite array is enriched with 16-mors (**Figure 5**A and B). Unlike the abovementioned two samples, a region dominated by a 20-mor appears to the left of the 16-mor region in the CHM13 satellite array (Figure 5C). These results suggest that different levels of LN-HORs occur in human centromeres, and that with subsequent mutations these may ultimately contribute to HOR landscape differentiation.

## Discussion

In this study, we investigated the population diversity of human centromere sequences on the basis of both HiFi reads and haplotype-resolved assemblies of hundreds of samples from two pangenome sequencing consortiums (HPRC and CPC). We reported considerable diversity in centromere HOR array size for different samples on the basis of HiFi read estimation, with CHM13 representing only one of many possible human HOR patterns. In addition, 33 HORs presented variable numbers across all the samples, 23 of which presented significantly different distributions among the three populations. We detected three centromere genotypes with unbalanced population frequencies on Chr5, Chr8, and Chr17. Moreover, a comparative analysis of satellite arrays across assemblies revealed that although human centromere HOR array structures are diverse, they nevertheless tend to resolve into a relatively small number of landscapes, with LN-HORs playing an important role in their diversification. These observations are based on high-quality assemblies published by HPRC [14] and CPC [13]. The HOR landscapes reported in our study represent the common features of multiple samples. Misassembly may affect the quantification of different landscapes. Future complete assemblies will refine a more accurate quantification for different landscapes.

In a previous analysis of the b/n boxes (CENP-B-binding site and pJα protein-binding site) within the HOR units of a single genome, a cyclical model was proposed to explain how HOR units vary among chromosomes and are structured around three states with short, moderately longer, and substantially longer HOR units, respectively [19,20]. Under this model, the rate of tandem expansion within longer HOR units is higher than that within shorter HOR units, but shorter HOR units have a more stable b/n box structure and thus can better resist invasion by pericentromeric heterochromatin. This degree of competition between the rate of tandem expansion (which determines the HOR length) and its capacity to resist invasion (which determines b/n box completeness) influences which HOR expands, and how greatly.

**Figure 6:**
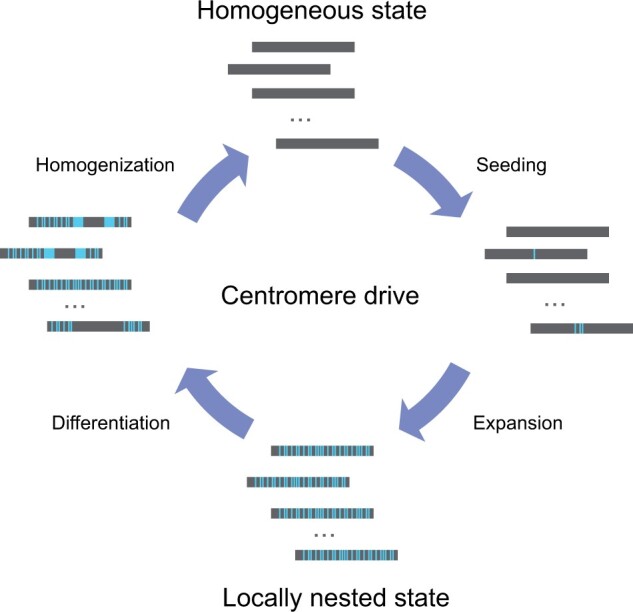
Cyclical model of HOR landscape evolution in the human population In the homogeneous state, all HOR arrays are homogeneous with only one HOR large expansion. In the seeding stage, variation occurs in the original HORs forming the LN-HOR units (seeding stage). In the expansion stage, under centromere drive [21], LN-HOR units expand rapidly and centromeres with a large number of LN-HOR units spread in the population, forming a locally nested state. In the differentiation stage, HORs with different monomer lengths and b box contents compete in the array, leading to different or even dramatic changes in HOR landscapes. Finally, one type of HOR unit may "win" the competition and gradually return to a homogeneous state (homogenization stage). LN-HOR, locally nested HOR.

However, how do centromere HOR arrays evolve in the broader human population? In this study, on the basis of the analysis of hundreds of human genomes, we reported considerable diversity of HOR landscapes for each chromosome which may be explained by a similarly cyclical model centered on the transition of HOR landscapes between homogeneous and locally nested states (**Figure 6**). In a homogeneous landscape, the centromere is dominated by a single HOR, such as that observed on Chr19, Chr22, and ChrX. The recurrent expansion of these HOR subunits eventually “breaks” the homogeneous landscape (through replication errors such as monomer duplication), forming locally nested units that have the advantage of a relatively high expansion rate (due to disproportionately occurring in the middle of existing HORs, they are protected from invasion by pericentromeric heterochromatin). These newly expanded subunits may increase the density of the b box, similar to our observations on Chr11 landscape 2 (Figure S29A), obtaining a competitive advantage relative to the homogeneous state (we call this the “seeding stage”). Following centromere drive [21], LN-HORs rapidly expand and thus spread throughout the population (“expansion stage”). These two stages may be typified by Chr3, Chr11, Chr12, Chr14, and Chr20, each of which has dual-landscape centromeres, one being highly homogeneous and the other with varying levels of local nesting. LN-HOR expansion results in a state with high LN-HOR content in the array and a high LN centromere ratio in the population, represented by Chr1, Chr2, Chr9, and Chr15 (“locally nested state”). Once a locally nested state is formed, the entire array is composed of original HORs, the LN subunits, and their combinations with different levels. These HOR units containing different monomer lengths and b box contents may achieve different advantages for competition, leading to rapid and pronounced changes in HOR landscapes, such as Chr5 and Chr8 (“differentiation stage”). Ultimately, one type of HOR unit may “win” in competition and gradually homogenize the landscape (“homogenization stage”), whereupon the cycle repeats.

Three HOR landscapes on Chr10 may record this cycle (Figure 4C). Landscape 1 (*n* = 6 samples) consists entirely of 10_M2L8, representing the homogeneous state. In landscape 2 (*n* = 59 samples), 10_M2L8 and 10_M1L6 (subunit of 10_M2L8) coexist across the entire satellite array, representing a locally nested state. For landscape 3 (*n* = 44 samples), a significant expansion of 10_M1L6 is observed in the middle of the array with the co-occurrence of M1L6 and M2L8 on either side, which may represent a period of transition from a locally nested to a homogeneous state. We observed a high density of b boxes in the newly expanded 10_M1L6 on landscape 3, which may also provide support for the presumed selective advantage of this state (Figure S29B).

In summary, using a large number of samples with high-coverage HiFi reads data and high-quality haplotype-resolved assemblies, we demonstrated the diversity of human centromere HOR patterns and explored how they evolved. In the future, the release of more human genomes and the accumulation of related epigenetic data have the potential to refine this model, and so may provide further insights into our understanding of the mechanisms of centromere evolution.

## Materials and methods

### HiFi sequencing data and genome assembly

We obtained HiFi reads for 43 samples released by the HPRC [14], 58 samples by the CPC [13], plus CHM13 [2]. The accession numbers and sample metadata for both the HiFi reads and the assemblies are given in Table S1. All HiFi reads data were first converted into fasta format via seqtk (v1.3-r106; https://github.com/lh3/seqtk). SAMtools (v1.12) was used to calculate the read length for each sample [22]. We also obtained 22 contig-level haplotype-resolved assemblies (11 samples) from the CPC and 86 assemblies (representing 43 individuals) from the HPRC and CHM13 genomes [2,13,14]. For each contig-level assembly, we used RagTag (v2.1.0) [23] compared with the CHM13 genome to generate the chromosome-level assembly. For each HPRC assembly, haplotypes 1 and 2 (H1 and H2) represent the paternal and maternal assemblies, respectively.

### Building chromosome-specific alpha satellite arrays

For each chromosome of each assembly, alpha satellite units were identified by using Lastz (v1.04.15) [16] to map the alpha satellite template sequence. Alpha satellite units mapped < 5 kb from each other were merged to produce a set of alpha satellite regions, and the regions with total length < 10 kb were discarded. Finally, for each chromosome, we concatenated its set of alpha satellite regions, producing its satellite array.

### ASR classifier training

HiCAT-human-reads contains a two-step classifier based on principal component analysis (PCA) and support vector machine (SVM) for extracting chromosome-specific ASRs. The first step involved detecting ASRs, and the second step involved assigning ASRs to their chromosomes of origin. First, a training dataset was simulated using the CHM13 genome with HG002 ChrY (CHM13-HG002 chrY) [2,24]. To simulate ASRs, satellite arrays from CHM13-HG002 ChrY were randomly broken into reads with lengths of 10−25 kb, of which we reversed half of the set to represent the “−” strand. We repeated the simulation 10 times. To ensure a balanced dataset, the number of ASRs simulated from each chromosome was equal. We simulated the same number of negative (non-ASR) samples for training by randomly sampling 10−25-kb subsequences from CHM13-HG002 ChrY, with the exception of the alpha satellite regions. The simulated ASRs were labeled “Alpha” and the non-ASRs were labeled “Non-Alpha” for the first step, with the former labeled according to their corresponding chromosomes for the second step. Second, we calculated the frequency of all 5-mers to construct a 5-mer frequency matrix $M_{n\times m}$ for all simulated reads, where n denotes the read count and m=512 denotes all 5-mers on each of the two (“+/−”) strands (1024/2=512). We normalized the 5-mer frequency matrix by the corresponding read length and then used PCA to reduce the dimension of the normalized 5-mer frequency matrix while ensuring that the amount of variance it explained exceeded 95%. Finally, we adopted an SVM to classify the reads.

### Running HiCAT and monomer template selection

We ran HiCAT (v1.1) [15] on each satellite array from CHM13-HG002 ChrY to annotate its monomers and HORs. For each satellite array, HiCAT annotates its constituent monomers via a community detection approach [25]. In brief, for a monomer community with more than one monomer, HiCAT calculates pairwise edit distances between monomers and then selects the monomer sequence with the lowest edit distance to all other monomers in the community as its output (that is, the template sequence from which HORs are built).

### HOR annotation

For HiCAT-human annotation, we first used the StringDecomposer algorithm [17] to decompose the ASRs and satellite arrays into block sequences. Then, for each chromosome, we calculated the sequence identity between each block and the monomer template (obtained by HiCAT annotation of CHM13-HG002 ChrY, described above). The sequence identity was defined as follows:

(1) $$\mathrm{identity}=\frac{ed(s_{\mathrm{block}},s_{\mathrm{monomer}})}{\max(l_{\mathrm{block}},l_{\mathrm{monomer}})}$$

where $ed(s_{\mathrm{block}},s_{\mathrm{monomer}})$ is the edit distance between the block and monomer template sequence, $l_{\mathrm{block}}$ is the sequence length of the block, and $l_{\mathrm{monomer}}$ is the sequence length of the monomer template. We labeled the block with the largest identity monomer ID to transform the block sequences into monomer sequences. We classified blocks whose highest identity was lower than 90% as ‘unknown sequences’; this may represent transposable elements or other sequences interjected into the HOR. Since LN-HORs occur in human centromeres, HiCAT-human adopted the HTRM method which we previously developed [15], to detect and compress local tandem repeats in the monomer sequences recursively to obtain HORs in each chromosome. This ensures that HORs with shifted or reversed units, such as those with MPs of 1 − 2−3 − 4, 4 − 1−2 − 3, 3 − 4−1 − 2, 2 − 3−4 − 1, and 4 − 3−2 − 1, are grouped together via the same HOR. For presentation purposes, HORs were sorted on the basis of their total number of repeats and then named following the convention “R + rank + L + unit monomer length”.

### Multisample HOR aggregation

To compare cross-sample annotations, we aggregated the annotation results for both the HiFi reads and the assemblies. For HiFi reads, HORs with shifted and reversed units were grouped together with the same HOR pattern (as described above). HOR patterns were sorted by the total repeat number across all samples and named following the convention “M + rank + L + unit monomer length”. For assembly, HOR pattern names are matched with HiFi reads.

### Quantification of HiFi read HORs among all samples

The estimated HOR array size $s_{i,k}$ of chromosome i in sample k is defined as:

(2) $$s_{i,k}=\frac{l_{i,k}}{c_{k}}$$

where $l_{i,k}$ is the total length of HiFi reads with HORs of chromosome i in sample k, and $c_{k}$ is the sequencing coverage of sample k.

The n-number $n_{j,k}$ of HOR j in sample k is calculated as follows:

(3) $$n_{j,k}=\frac{r_{j,k}}{c_{k}}$$

where $r_{j,k}$ represents the number of HOR j in sample k output by HiCAT-human-reads. We excluded rare HORs that met the following conditions:

(4) $$\forall k,n_{j,k}<0.1\times \sum_{j\in I_{j,k}}n_{j,k}$$

where $I_{j,k}$ represents all HORs on HOR j corresponding chromosome of sample k. For the remaining HORs, the mean fold change $mf_{j,k}$ of HOR j in sample k is calculated as follows:

(5) $$mf_{j,k}=\frac{n_{j,k}}{\mathrm{avg}n_{j}}$$

where $\mathrm{avg}n_{j}$ is the mean number of HOR j among samples. Then, we calculated the standard deviation $std_{j}$ of the mean fold change in the HOR j among samples. The v-HOR is defined as an HOR j with $std_{j}$ greater than 0.5. We obtained 33 v-HORs.

### Cross-landscape HOR clustering

To compare the HORs among different landscapes, we performed a cross-landscape HOR clustering analysis (Figure S30). For different landscapes, sequences of the largest MP which their primary HORs shared were extracted. For example, on Chr5, the primary HORs are M3L12 on landscape 1 and M2L8 on landscape 2. Since M2L8 is a subunit of M3L12, we selected M2L8 as the target HOR on Chr5 for cross-landscape HOR clustering. We extracted all the DNA sequences of the target HOR units in different satellite arrays and performed multiple alignments for these sequences via Kalign (v3.3.5) [26]. The most common base at every position of the alignment files was subsequently identified to construct the target HOR consensus sequence. All extracted HOR DNA sequences were pairwise aligned with the consensus sequence (needle, EMBOSS v6.6.0) [27] and reformatted into 0−1 vectors, where 0 indicates that the HOR shares the same base with the consensus sequence at that position and 1 indicates that there is a difference. The 0−1 vectors of all HOR units were clustered on the basis of *k*-means. For each target HOR, we chose the smallest *k* that could represent the difference among landscapes.

### Ancestral HOR sequence reconstruction

To explore the evolution of HOR clusters among different landscapes, we reconstructed ancestral HOR sequences and compared the HOR cluster consensus sequences with ancestral HOR sequences. For each target chromosome, we used monomers from another chromosome in the same suprachromosomal family (SF) as the outgroup to reconstruct the ancestral HOR sequence [12]. For Chr5 and Chr10 (SF1), we used monomers from Chr19 since its primary HOR has the same unit monomer length as the ancestor, and for Chr11 (SF3), we used Chr17. For each outgroup chromosome, we generated the consensus sequence of each monomer of the primary HOR and constructed a monomer phylogenetic tree via IQ-TREE (v2.2.5) [28]. The monomers were divided into groups and the group number was based on the SF ancestral HORs. We calculated the consensus sequences of the monomers from each group as ancestral monomers. Then, for each target chromosome, we generated the consensus sequence of each monomer in the target HOR. We used these sequences with the ancestral monomers to construct a phylogenetic tree via IQ-TREE, which revealed the correspondence between the ancestral monomers and target monomers. We then derived the ancestral HOR DNA sequence on the basis of ancestral monomers and targeted HOR. Finally, we used Clustal Omega [29,30] to perform multiple sequence alignment of the ancestral sequence with the consensus sequences of each HOR cluster, inferring their relationship from the identity matrix.

## Code availability

HiCAT-human code has been deposited at GitHub (https://github.com/xjtu-omics/HiCAT-human), BioCode at the National Genomics Data Center, Beijing Institute of Genomics, Chinese Academy of Sciences / China National Center for Bioinformation (BioCode: BT007545, https://ngdc.cncb.ac.cn/biocode/tools/BT007545), and Zenodo (https://zenodo.org/doi/10.5281/zenodo.10570850). The cross-landscape HOR clustering analysis scripts have been deposited at GitHub (https://github.com/xjtu-omics/Cross_landscape_HOR_clustering) and Zenodo (https://zenodo.org/doi/10.5281/zenodo.10570634).

## Supplementary Material

qzae071_Supplementary_Data

## Data Availability

The HOR annotation data generated in this study are available in FigShare at https://figshare.com/articles/dataset/HiFi_reads_and_assemblies_HOR_annotation/25067558.

## CRediT author statement

**Shenghan Gao:** Conceptualization, Methodology, Software, Validation, Formal analysis, Investigation, Data curation, Writing – original draft, Visualization. **Yimeng Zhang:** Software, Validation, Formal analysis, Investigation, Visualization. **Stephen J. Bush:** Writing – original draft. **Bo Wang:** Investigation, Funding acquisition. **Xiaofei Yang:** Conceptualization, Methodology, Writing – original draft, Supervision, Funding acquisition. **Kai Ye:** Conceptualization, Supervision, Funding acquisition. All authors have read and approved the final manuscript.
