# Supplementary material for: Centromere Landscapes Resolved from Hundreds of Human Genomes
Source: Genomics Proteomics Bioinformatics. 2024 Oct 18;22(5):qzae071. doi: 10.1093/gpbjnl/qzae071 (PMC11652271; doi:10.1093/gpbjnl/qzae071)
Supplement: qzae071_Supplementary_Data [file qzae071_supplementary_data.zip › Figure S21.pdf]

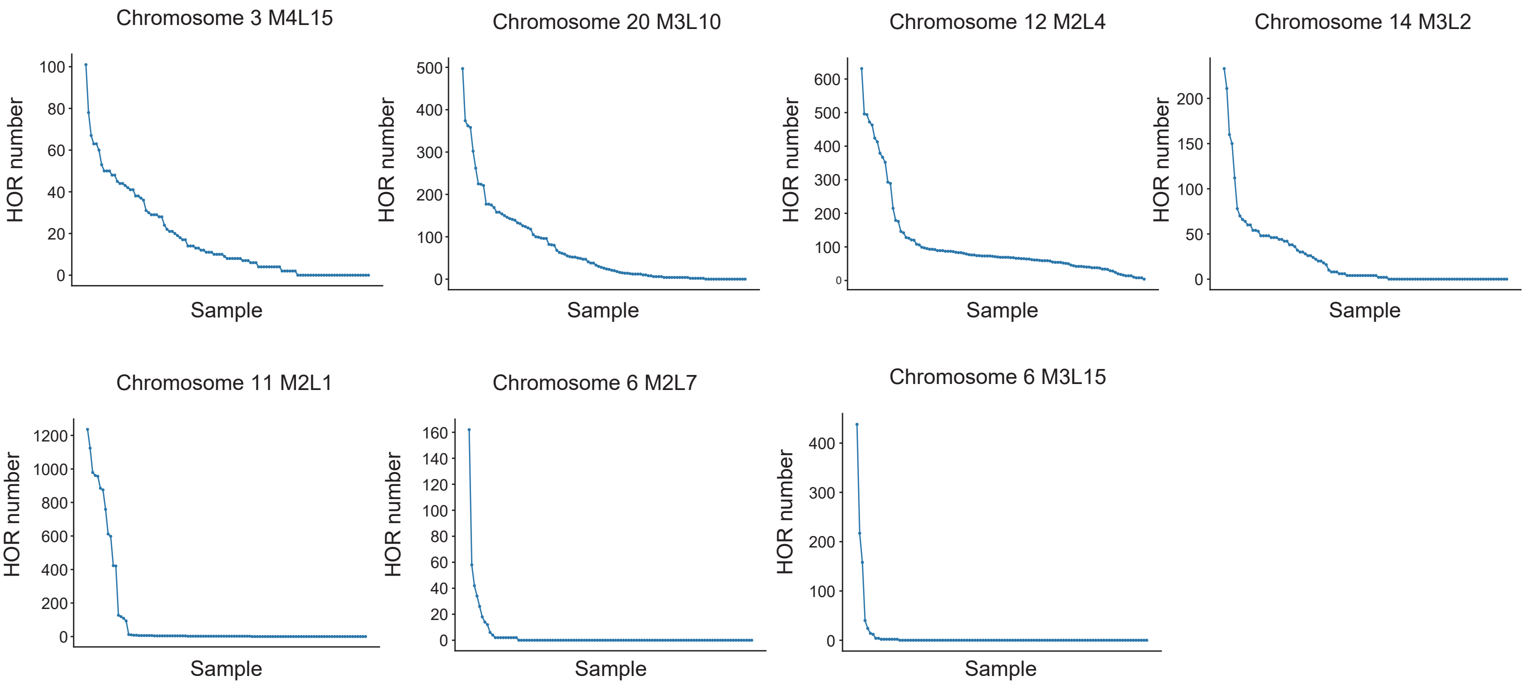
Chromosome 3 M4L15
HOR number
100
80
60
40
20
0
Sample
Chromosome 20 M3L10
HOR number
500
400
300
200
100
0
Sample
Chromosome 12 M2L4
HOR number
600
500
400
300
200
100
0
Sample
Chromosome 14 M3L2
HOR number
200
150
100
50
0
Sample
Chromosome 11 M2L1
HOR number
1200
1000
800
600
400
200
0
Sample
Chromosome 6 M2L7
HOR number
160
140
120
100
80
60
40
20
0
Sample
Chromosome 6 M3L15
HOR number
400
300
200
100
0
Sample
