# Supplementary material for: Centromere Landscapes Resolved from Hundreds of Human Genomes
Source: Genomics Proteomics Bioinformatics. 2024 Oct 18;22(5):qzae071. doi: 10.1093/gpbjnl/qzae071 (PMC11652271; doi:10.1093/gpbjnl/qzae071)
Supplement: qzae071_Supplementary_Data [file qzae071_supplementary_data.zip › File S1.docx]

**File S1 Simulation test for reads classification**

To examine the performance of the HiCAT-human-reads workflow, we simulated 30× HiFi sequencing data of the CHM13 genome as a basic test, and chromosome Y was from the HG002 genome. The simulated sequencing error rate was 0.1%, and the read length ranged from 10 kb to 25 kb [1]. We repeated the simulation five times. We first evaluated the classification accuracy of all the simulated alpha satellite reads. We found that the average recall was 87.8% (Figure S1A; Table S2). We found that most of falsely classified ASRs were classified into non-alpha regions (Figure S1B). We further investigated the locations of falsely classified ASRs and found that they were concentrated in the flanking active HOR regions (Figure S1C). Chromosomes 13, 14, 15, and 21 each had a recall lower than 75%, which we found was driven by the false classification of ASRs in chromosomes 13, 14, and 15 as chromosome 21 due to segmental duplications (Figure S1D). We summarized the HOR annotations of falsely classified ASRs and reported that the average HOR coverage ratio (percent of HOR bases covered by a read) was only 4.3% and that 75.83% of the falsely classified ASRs did not have HORs, indicating that these reads have less impact on further HOR analysis (Table S3). We subsequently examined the classification recall of simulated reads from active HOR regions defined in previous studies [2] and reported that the average recall was 99.7% (Figure S1E; Table S2). We found that most falsely classified reads in active HOR regions were found on chromosomes 1, 13, 14, and 22 because chromosomes 1/5/19, 13/21, and 14/22 each contained a shared set of HORs [2] and were located in their marginal areas, indicating that the marginal areas of active HOR regions were more similar across chromosomes (Figure S1F).

**References**

[1] Wenger AM, Peluso P, Rowell WJ, Chang PC, Hall RJ, Concepcion GT, et al. Accurate circular consensus long-read sequencing improves variant detection and assembly of a human genome. Nat Biotechnol 2019;37:1155−62.

[2] Altemose N, Logsdon GA, Bzikadze AV, Sidhwani P, Langley SA, Caldas GV, et al. Complete genomic and epigenetic maps of human centromeres. Science 2022;376:eabl4178.
