# Supplementary material for: Centromere Landscapes Resolved from Hundreds of Human Genomes
Source: Genomics Proteomics Bioinformatics. 2024 Oct 18;22(5):qzae071. doi: 10.1093/gpbjnl/qzae071 (PMC11652271; doi:10.1093/gpbjnl/qzae071)
Supplement: qzae071_Supplementary_Data [file qzae071_supplementary_data.zip › supplementary material captions.docx]

**Supplementary material**

**File S1 Simulation test for reads classification**

**Figure S1** **Reads classification evaluation of simulated HiFi sequencing data**

**A.** Recall of reads classification in all alpha satellite regions for each chromosome. **B.** Heatmap of falsely classified reads in all alpha satellite regions normalized on the basis of z-score. **C.** The location and level of falsely classified reads in all alpha satellite regions. The window size is 500 kb. **D.** Dot plots between CHM13 chromosome 21 satellite arrays and chromosomes 13, 14, and 15. **E.** Recall of reads classification in active HOR regions for each chromosome. **F.** The location of falsely classified reads in active HOR regions. CEN indicates active HOR regions in each chromosome centromere.

**Figure S2**  **HOR quantification on chromosome X and chromosome Y**

**A.** Estimating HOR array size on the basis of total HOR read length and sequencing coverage. **B.−C.** The variation in the HOR mean fold change on chrX (B) and chrY (C) among all the samples. CHM13 is represented by red, and the other samples are gray. v-HORs are marked by stars.

**Figure S3**  **Chromosome HOR array size in AFR, AMR, and EAS was significantly greater in EAS than in other populations**

The HOR array size is significantly larger in the EAS samples than in both the AFR samples and AMR samples on chromosomes 2, 4, 6, 7, 9, 10, 13, and 15. *P* value is calculated via a one-sided Wilcoxon rank sum test.

**Figure S4** **Chromosome HOR array size in AFR, AMR and, EAS populations was significantly greater than that in other populations**

The HOR array size is significantly larger in the AFR samples than in both EAS samples and AMR samples on chromosomes 16, 21, and Y. *P* value is calculated via a one-sided Wilcoxon rank sum test.

**Figure S5**  **Chromosome HOR array sizes for AFR, AMR, and EAS on other chromosomes**

*P* value was calculated via a one-sided Wilcoxon rank sum test.

**Figure S6**  **v-HORs showing significant variance among populations**

*P* value was calculated via a two-sided Wilcoxon rank sum test.

**Figure S7** **v-HORs not showing significant variance among populations**

*P* value was calculated via a two-sided Wilcoxon rank sum test.

**Figure S8** **Spearman correlation between all HORs**

**A.** Heatmap showing the correlation of HOR n-numbers. The different colors in the left bar indicate the HOR from different chromosomes. **B.** Absolute value of the n-number Spearman correlation coefficient of inter- and intra-chromosome HORs. *P* value was calculated via a one-sided Wilcoxon rank sum test.

**Figure S9 The PCA results of sample clustering using n-numbers of HORs on chr5 (A) and chr8 (B)**

**Figure S10** **Sample clustering based on HORs in chr3 and chr4**

**A.** The heatmap and sample hierarchical clustering of HOR n-numbers in chromosome 3. **B.** The PCA result of sample clustering using HOR n-numbers in chr3. **C.** Monomer patterns of 3_M1L17, 3_M4L15, and 3_M2L10. **D.** The proportion of samples in each of the AFR, AMR, and EAS populations containing 3_C0, 3_C1, and 3_C2. **E.** The heatmap and sample hierarchical clustering of HOR n-numbers in chromosome 4. **F.** The PCA result of sample clustering using HOR n-numbers in chr4. **G.** Monomer patterns of 4_M1L19, 4_M2L15, and 4_M3L13. **H.** The proportion of samples in each of the AFR, AMR and EAS populations containing 4_C0, 4_C1, and 4_C2.

**Figure S11 Sample clustering based on HORs in chr6 and chr7**

**A.** The heatmap and sample hierarchical clustering of HOR n-numbers in chromosome 6. **B.** The PCA result of sample clustering using HOR n-numbers in chr6. **C.** Monomer patterns of 6_M1L18, 6_M2L7, 6_M3L15, and 6_M4L2. **D.** The proportion of samples in each of the AFR, AMR, and EAS populations containing 6_C0, 6_C1, and 6_C2. **E.** The heatmap and sample hierarchical clustering of HOR n-numbers in chromosome 7. **F.** The PCA result of sample clustering using HOR n-numbers in chr7. **G.** Monomer patterns of 7_M1L6, 7_M3L10, and 7_M5L2. **H.** The proportion of samples in each of the AFR, AMR, and EAS populations containing 7_C0 and 7_C1.

**Figure S12** **Sample clustering based on HORs in chr10 and chr11**

**A.** The heatmap and sample hierarchical clustering of HOR n-numbers in chromosome 10. **B.** The PCA result of sample clustering using n-numbers of HORs in chr10. **C.** Monomer patterns of 10_M1L6, 10_M2L8, and 10_M4L2. **D.** The proportion of samples in each of the AFR, AMR, and EAS populations containing 10_C0, 10_C1, and 10_C2. **E.** The heatmap and sample hierarchical clustering of HOR n-numbers in chromosome 11. **F.** The PCA result of sample clustering using HOR n-numbers in chr11. **G.** Monomer patterns of 11_M1L5 and 11_M2L1. **H.** The proportion of samples in each of the AFR, AMR and EAS populations containing 11_C0 and 11_C1.

**Figure S13**  **Sample clustering based on HORs in chr12 and chr13**

**A.** The heatmap and sample hierarchical clustering of HOR n-numbers in chromosome 12. **B.** The PCA result of sample clustering using HOR n-numbers in chr12. **C.** Monomer patterns of 12_M1L8 and 12_M2L4. **D.** The proportion of samples in each of the AFR, AMR and EAS populations containing 12_C0, 12_C1, and 12_C2. **E.** The heatmap and sample hierarchical clustering of HOR n-numbers in chromosome 13. **F.** The PCA result of sample clustering using HOR n-numbers in chr13. **G.** Monomer patterns of 13_M1L11, 13_M2L4, 13_M4L7, 13_M5L5, and 13_M3L10. **H.** The proportion of samples in each of the AFR, AMR, and EAS populations containing 13_C0, 13_C1, and 13_C2.

**Figure S14** **Sample clustering based on HORs in chr14 and chr16**

**A.** The heatmap and sample hierarchical clustering of HOR n-numbers in chromosome 14. **B.** The PCA result of sample clustering using HOR n-numbers in chr14. **C.** Monomer patterns of 14_M1L8, 14_M2L1, and 14_M3L2. **D.** The proportion of samples in each of the AFR, AMR, and EAS populations containing 14_C0 and 14_C1. **E.** The heatmap and sample hierarchical clustering of HOR n-numbers in chromosome 16. **F.** The PCA result of sample clustering using HOR n-numbers in chr16. **G.** Monomer patterns of 16_M1L10, 16_M3L6, 16_M4L6, and 16_M2L8. **H.** The proportion of samples in each of the AFR, AMR, and EAS populations containing 16_C0 and 16_C1.

**Figure S15** **Sample clustering based on HORs in chr18 and chr20**

**A.** The heatmap and sample hierarchical clustering of HOR n-numbers in chromosome 18. **B.** The PCA result of sample clustering using HOR n-numbers in chr18. **C.** Monomer patterns of 18_M1L12, 18_M2L6, 18_M3L10, 18_M4L2, 18_M5L4, 18_M6L10, and 18_M8L6. **D.** The proportion of samples in each of the AFR, AMR, and EAS populations containing 18_C0 and 18_C1. **E.** The heatmap and sample hierarchical clustering of HOR n-numbers in chromosome 20. **F.** The PCA result of sample clustering using HOR n-numbers in chr20. **G.** Monomer patterns of 20_M1L16, 20_M2L8, 20_M3L10, 20_M4L11, and 20_M6L2. **H.** The proportion of samples in each of the AFR, AMR, and EAS populations containing 20_C0 and 20_C1.

**Figure S16**  **Sample clustering based on HORs in chr21 and chrY**

**A.** The heatmap and sample hierarchical clustering of HOR n-numbers in chromosome 21. **B.** The PCA result of sample clustering using HOR n-numbers in chr21. **C.** Monomer patterns of 21_M1L11, 21_M2L4, and 21_M3L1. **D.** The proportion of samples in each of the AFR, AMR, and EAS populations containing 21_C0 and 21_C1. **E.** The heatmap and sample hierarchical clustering of HOR n-numbers in chromosome Y. **F.** The PCA result of sample clustering using HOR n-numbers in chrY. **G.** Monomer patterns of Y_M2L1, Y_M1L34, Y_M3L1, and Y_M4L36. **H.** The proportion of samples in each of the AFR, AMR and EAS populations containing Y_C0 and Y_C1.

**Figure S17**  **Centromere genotype from sample clustering based on HOR n-numbers in chromosome 17**

**A.** The heatmap and sample hierarchical clustering of HOR n-numbers in chromosome 17. **B.** Monomer patterns of 17_M2L16, 17_M4L15, and 17_M3L12. **C.** The box plot of HOR n-numbers in 17_C0 (AA), 17_C1 (BB), and 17_C2 (AB). For each HOR, the Mean(C0+C1) represents the pairwise mean n-numbers in 17_C0 and 17_C1. **D.** The proportion of samples in each of the AFR, AMR, and EAS populations containing 17_C0, 17_C1 and 17_C2. JP is Japanese population.

**Figure S18** **HOR landscapes on chromosomes 3, 4, 6, 7, 8, and 12**

HOR landscapes on chr3 represented by HG02622 H2 and HG00621 H1 (**A**); chr4 represented by CHM13 and HG01358 H1 (**B**); chr6 represented by HG00735 H1, CHM13, and RY02 H2 (**C**); chr7 represented by CHM13 and HG02572 H1 (**D**); chr8 represented by HG00438 H1, CHM13, HG01243 H2, and NA18906 H2 (**E**); and chr12 represented by RY03 H2 and CHM13 (**F**). The triangle similarity heatmaps are generated by StainedGlass.

**Figure S19**  **HOR landscapes on chromosomes 14, 16, 17, 18, and 20**

HOR landscapes on chr14 represented by HG00733 H2 and CHM13 (**A**), chr16 represented by CHM13 and HG03516 H2 (**B**), chr17 represented by CHM13 and HG01109 H2 (**C**), chr18 represented by CHM13, NA18906 H2, and HG02559 H2 (**D**), and chr20 represented by CHM13 and HG01123 H2 (**E**). The triangle similarity heatmaps are generated by StainedGlass.

**Figure S20** **Cross-landscape 11_M1L5 clustering of chromosome 11 satellite arrays for all samples**

Each track is the clustering result of a sample. The samples in each column have an increasing number of 11_M2L1 from top to bottom and the content of 11_M2L1 increases between columns from left to right.

**Figure S21** **LN-HOR number among samples**

The polylines show the LN-HOR number in different samples sorted from high to low on chr3, 20, 12, 14, 11, and 6. The local expansion rates differ greatly between chromosomes, appearing relatively high for chr11 but lower for chr3 and 20. LN-HOR, local nested higher order repeats.

**Figure S22 Cross-landscape 5_M2L8 clustering result of chr5 satellite arrays for all samples**

Each track is the clustering result of a sample. Most samples in landscape 1 are mainly composed of R0, and most samples in landscape 2 contain R1 in the middle and right of the satellite array.

**Figure S23 Cross-landscape 10_M1L6 cluster result of chr10 satellite arrays for all samples**

Each track is the clustering result of a sample. Most samples in landscape 1 are mainly composed of R0. In most landscape 2 satellite arrays, there is a small R0 region on the edge of the HOR region and most of the array is composed of mixed R1 and R2. In most landscape 3 satellite arrays, R3 is in the middle of the array.

**Figure S24 HOR landscapes on chr19, 22 and X**

The HOR landscapes on chr19 (**A**), chr22 (**B**), and chrX (**C**) are all represented by CHM13. All the three chromosomes have one homogenous landscape that contains only one primary HOR. The triangle similarity heatmaps are generated by StainedGlass.

**Figure S25 HOR landscapes on chromosomes 1, 2, 9, and 15**

The HOR landscapes on chr1 (**A**), chr2 (**B**), chr9 (**C**) and chr15 (**D**) all represented by CHM13. All the four chromosomes have one landscape that contains many LN-HORs. The triangle similarity heatmaps are generated by StainedGlass.

**Figure S26 Monomer sequences of HORs on chr1, 2, 9, 19, 22, X, and 15**

**Figure S27 1_M2L6 monomer pattern distributions in different samples**

**A.** Sample clustering based on the Pearson correlation coefficient of the 1_M2L6 monomer pattern length. The LN ratio is the number of local nested units compared with the total number of 1_M2L6 units. **B.−F.** The 1_M2L6 unit monomer length distributions of samples with 8-mer peaks represented by HIFI032513D (B), 10-mer peaks represented by RY07 (C) and HG01891 (D), 12-mer peaks represented by CHM13 (E) and 14-mer peaks represented by HG01109 (F). **G.** The sample ratios of the 8-mer peak, 10-mer peak, 12-mer peak, and 14-mer peak samples among the AFR, AMR, and EAS populations.

**Figure S28 5_M3L12 monomer pattern distributions in different groups**

5_M3L12 unit monomer length distributions of samples in different groups. Samples in group 1 are represented by NA19240 H2 (**A**). The samples in group 2 are represented by HG00438 H2 (**B**). The samples in group 3 are represented by CHM13 (**C**). **D.** The 12mor, 16mor, and 20mor M3L12 distributions of samples in groups 1, 2, and 3.

**Figure S29 b box density in different regions of the chromosome**

**A.** The b box density of the RY08 H1 chr11 centromere array. The M2L1 local nested region has a higher b box density than the flanking regions do. **B.** The b box density of the RY07 H1 chr10 centromere array. The medium-sized region with homogeneous M1L6 HORs presented a greater b box density than did the flanking regions. The density window number is 50.

**Figure S30 Cross-landscape HOR clustering workflow**

Target HOR DNA sequences for clustering are first extracted from satellite array monomer sequences on the basis of HiCAT-human-assembly annotation. Multiple sequence alignment is subsequently performed on HOR DNA sequences to generate HOR consensus sequences. Each HOR DNA sequence is compared with the consensus sequence to obtain a 0−1 vector, where 0 indicates that this HOR sequence shares the same base with the consensus sequence at that position, and 1 indicates that there is a difference. Finally, the 0−1 vectors of all HOR units are clustered via k-means. For each target HOR, we choose the smallest k that can represent the difference among landscapes.

**Table S1 Accession numbers and sample metadata for HiFi reads and assemblies**

**Table S2 Reads classification recall in the 30× HiFi simulation test**

**Table S3 HOR coverage ratios of error-classified reads for all alpha satellite regions**

**Table S4 Estimated HOR array size**

**Table S5 The normalized number of HORs among samples in autosomes**

**Table S6 The normalized number of HORs among samples on chromosome X**

**Table S7 The normalized number of HORs among samples on chromosome Y**

**Table S8 The mean fold changes in HORs among samples in autosomes**

**Table S9 The mean fold change in HORs among samples from chromosome X**

**Table S10 The mean fold change in HORs among samples from chromosome Y**

**Table S11 Standard deviation of the mean fold change in HORs among the samples**

**Table S12 Consensus of the 79 HORs**

**Table S13 The normalized numbers of 33 v-HORs among different populations**

**Table S14 Spearman correlations between all HORs.**

**Table S15 Sample clustering based on HORs on chromosomes with v-HORs**

**Table S16 HOR normalized number comparison of different clusters in the HiFi reads data for chr5, 8, and 17**

**Table S17 Identity between the reconstructed ancestral HOR sequence and the consensus HOR sequence in each cluster**

**Table S18 Monomer pattern sample distribution on chromosome 1 M2L6**

**Table S19 Distribution of 12mor, 16mor and 20mor M3L12 of samples in group 1, group 2, and group 3**
