# Supplementary figures and images for: Centromere Landscapes Resolved from Hundreds of Human Genomes
Source: Genomics Proteomics Bioinformatics. 2024 Oct 18;22(5):qzae071. doi: 10.1093/gpbjnl/qzae071 (PMC11652271; doi:10.1093/gpbjnl/qzae071)

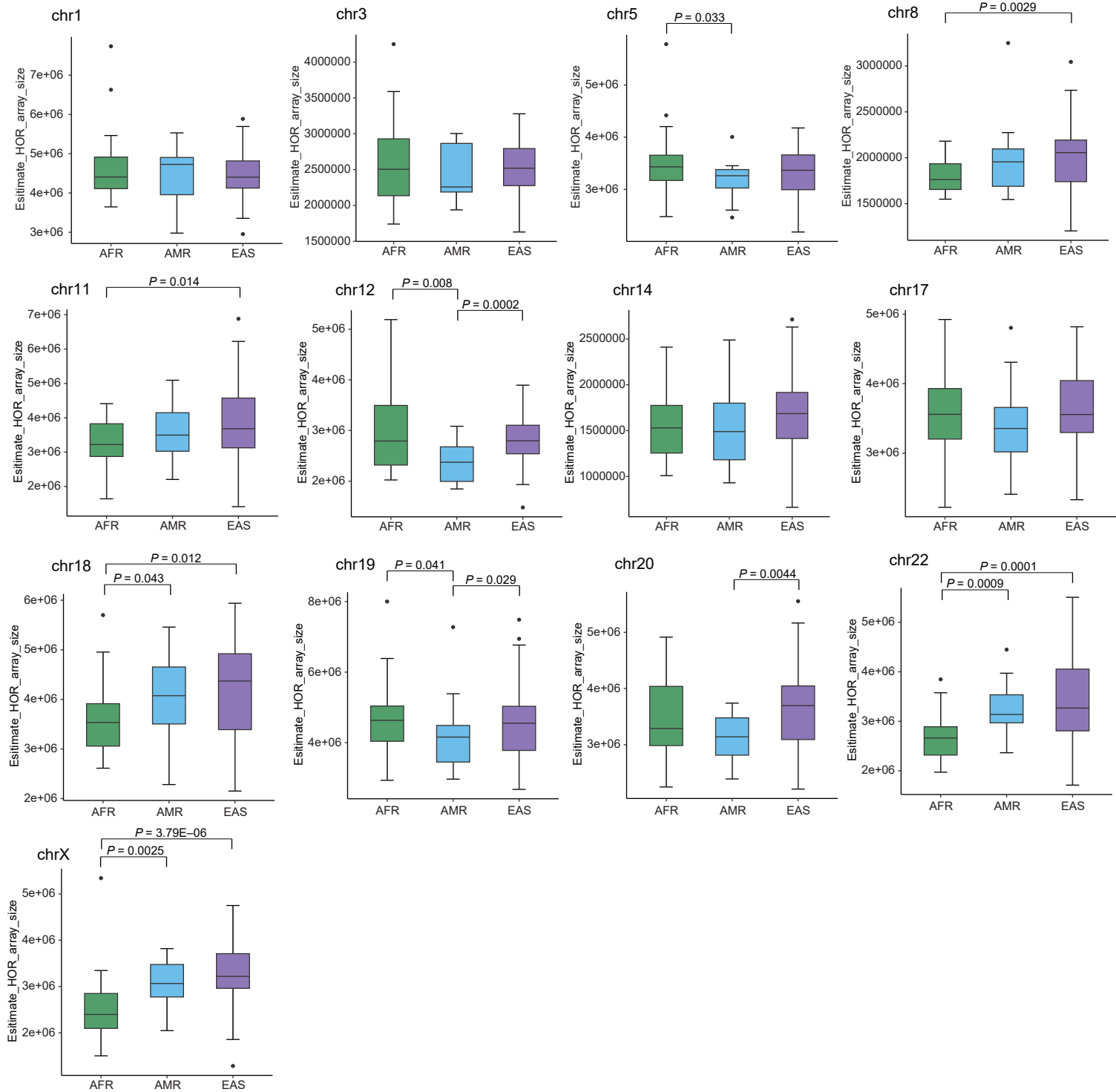

Supplement: qzae071_Supplementary_Data [file qzae071_supplementary_data.zip › Figure S5.pdf]

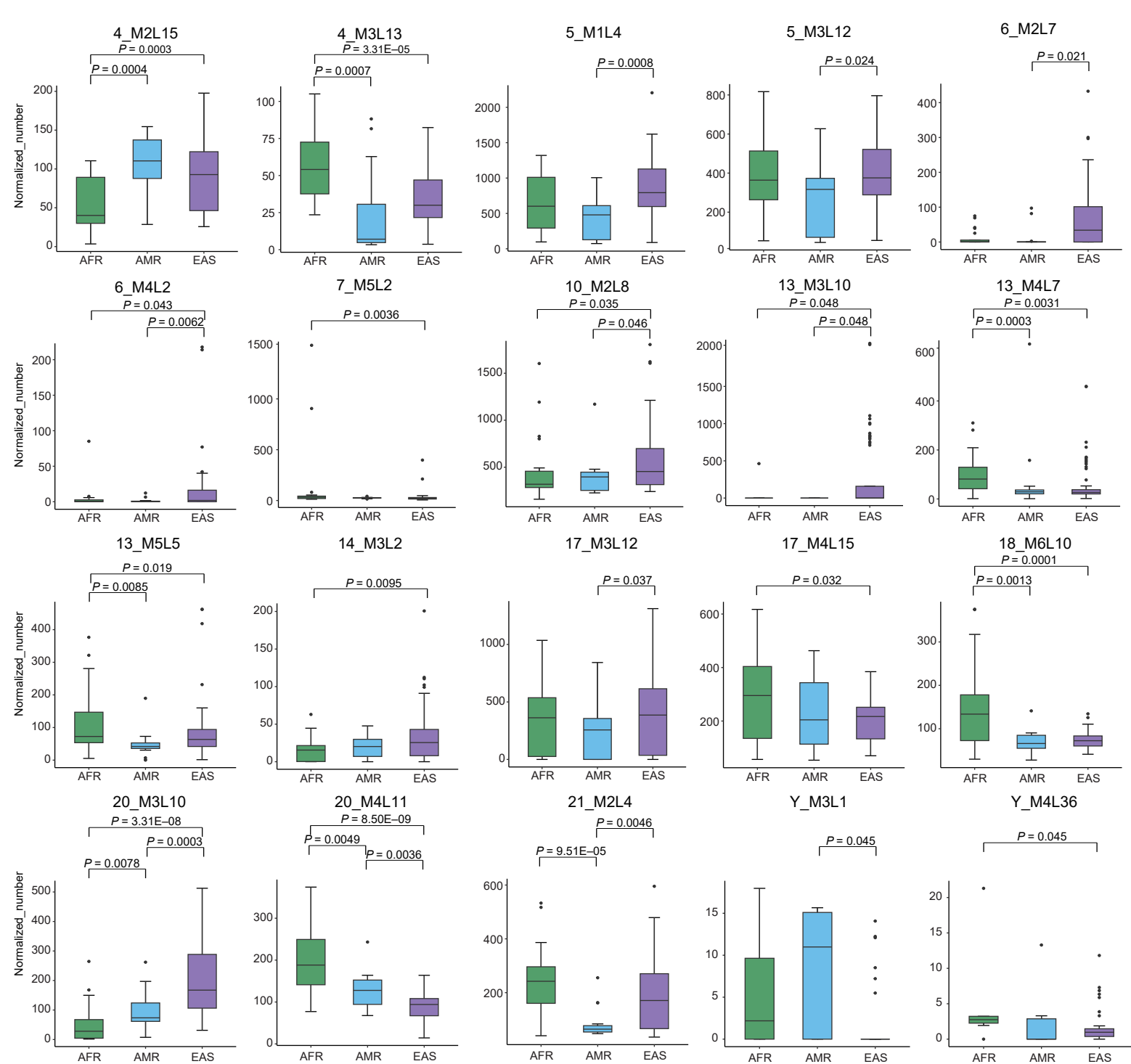

Supplement: qzae071_Supplementary_Data [file qzae071_supplementary_data.zip › Figure S6.pdf]

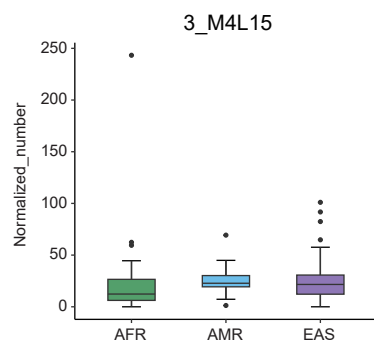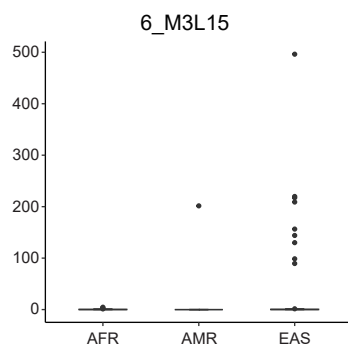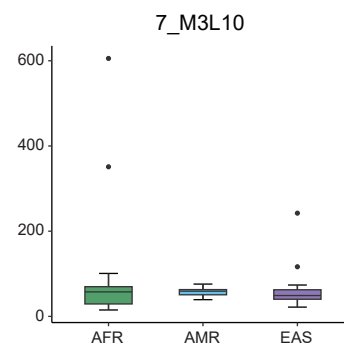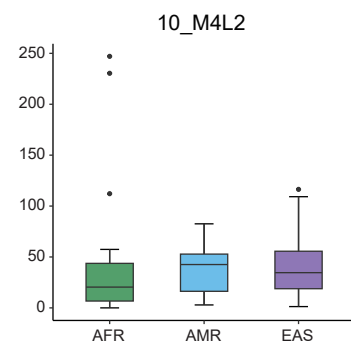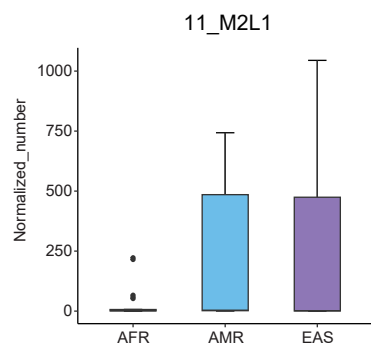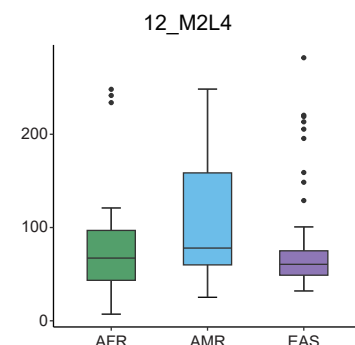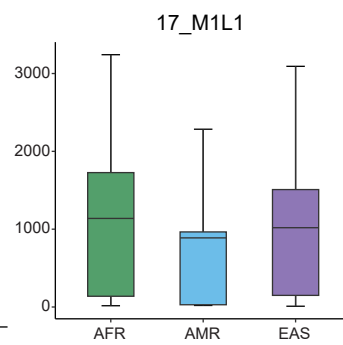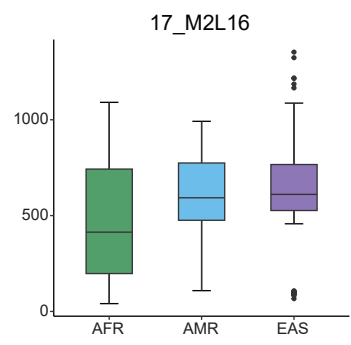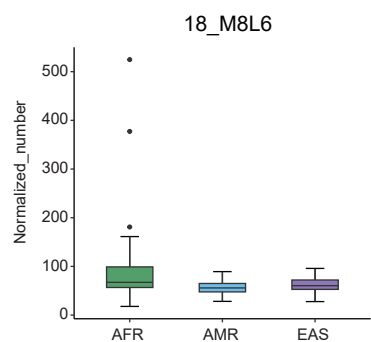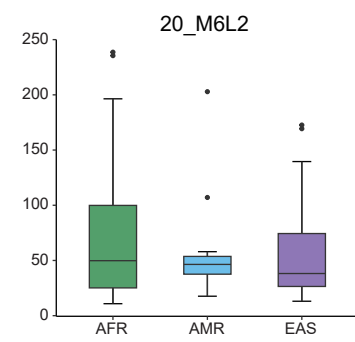

Supplement: qzae071_Supplementary_Data [file qzae071_supplementary_data.zip › Figure S7.pdf]

A

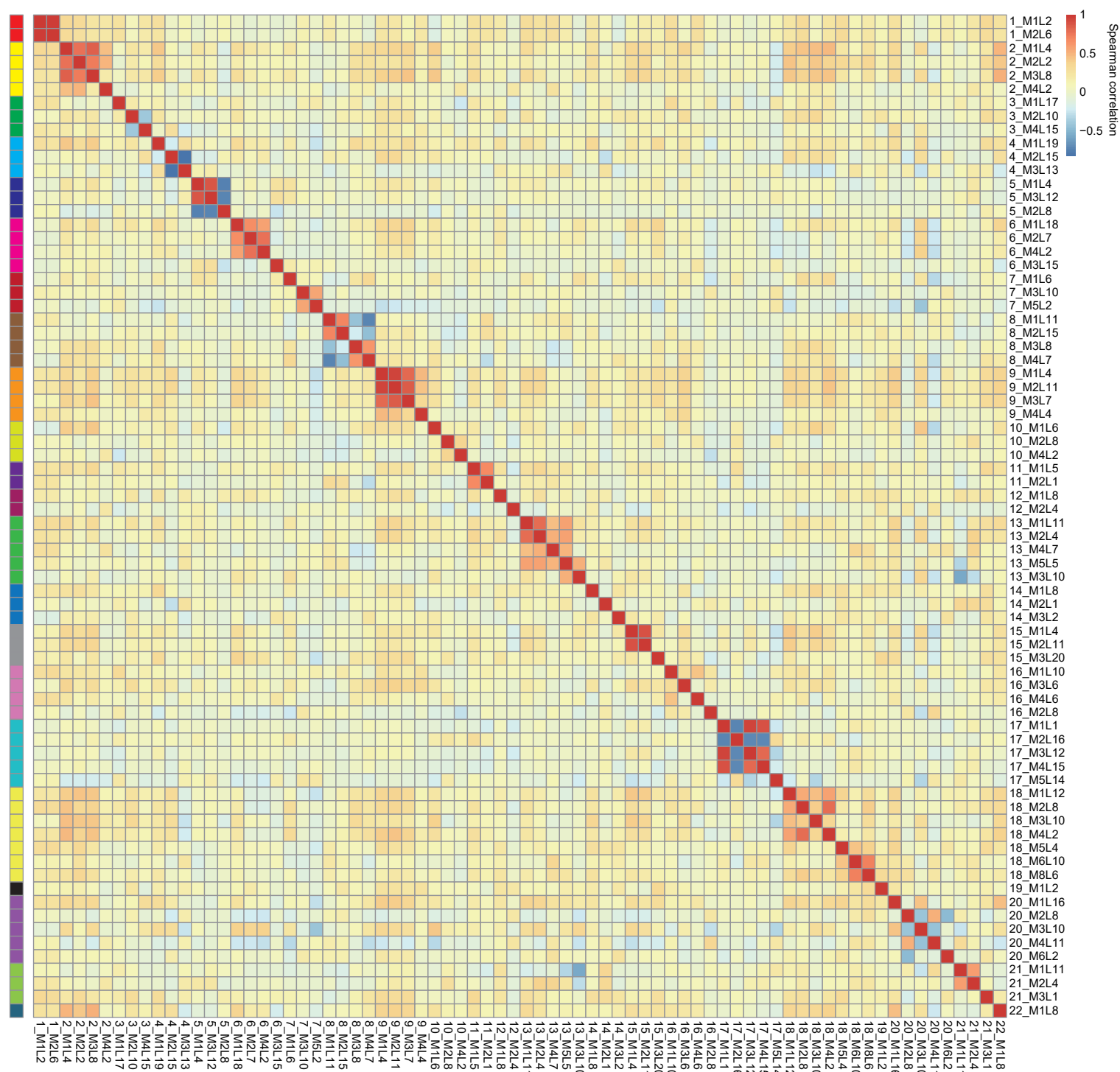

B

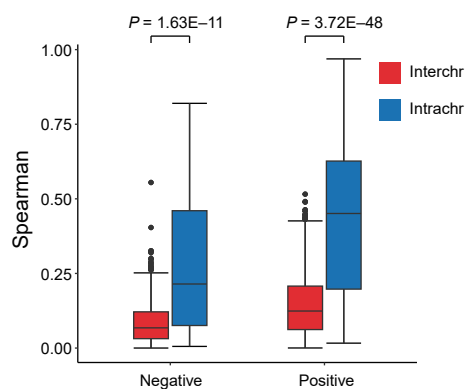

Supplement: qzae071_Supplementary_Data [file qzae071_supplementary_data.zip › Figure S8.pdf]

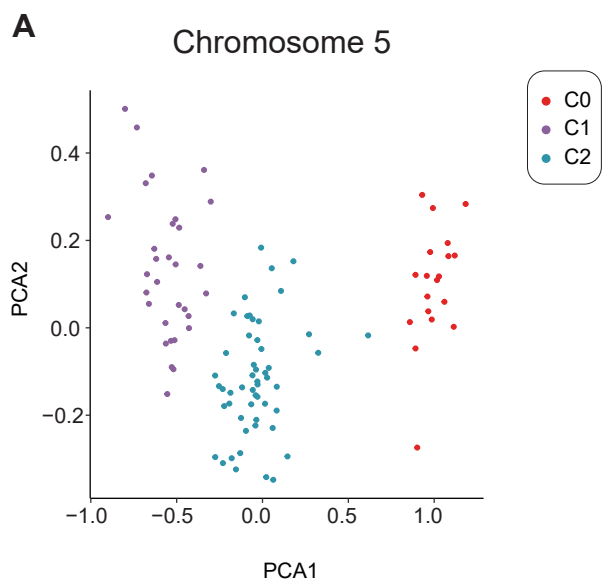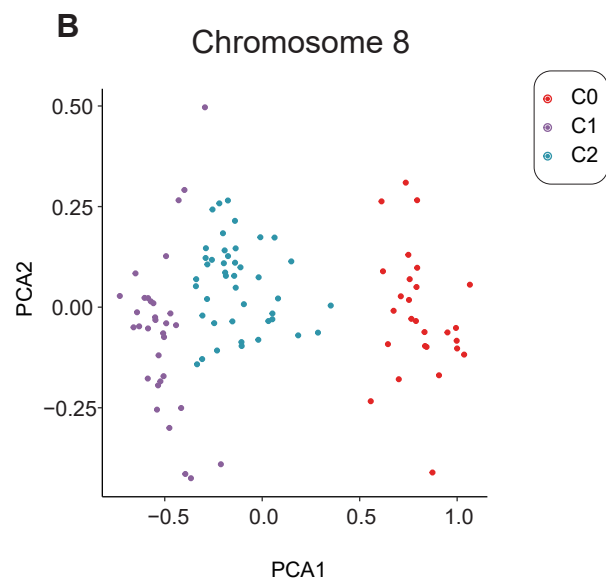

Supplement: qzae071_Supplementary_Data [file qzae071_supplementary_data.zip › Figure S9.pdf]

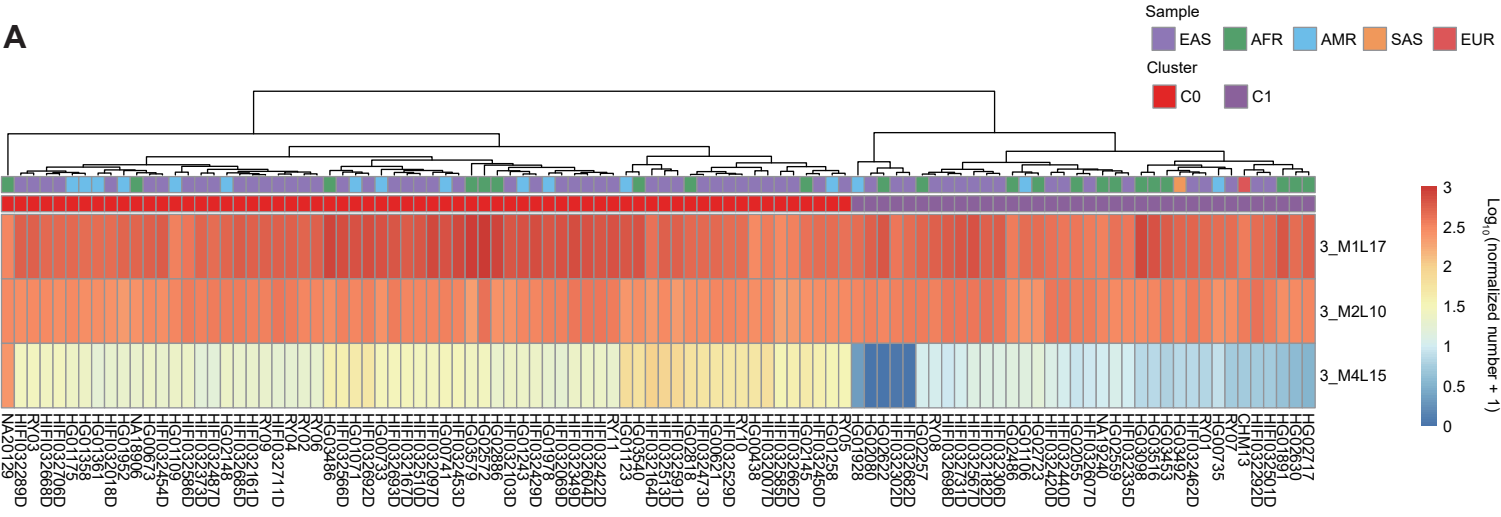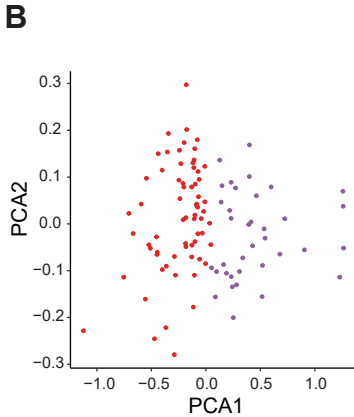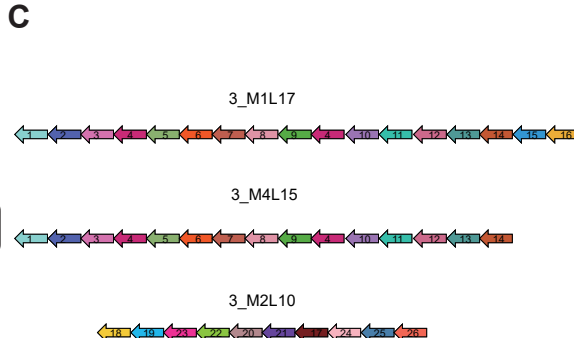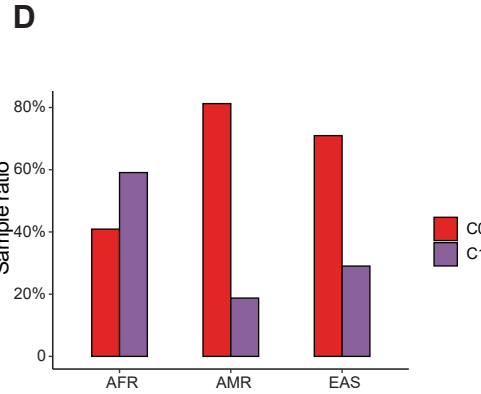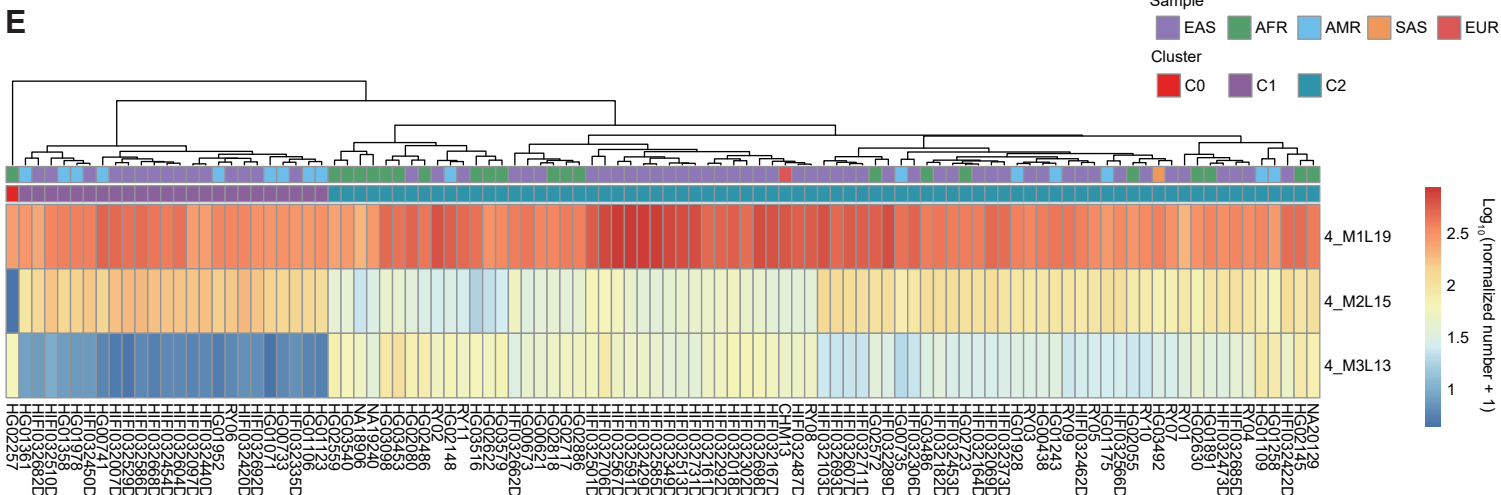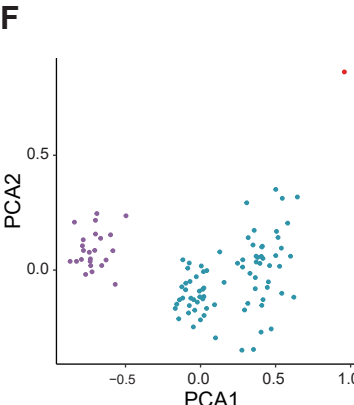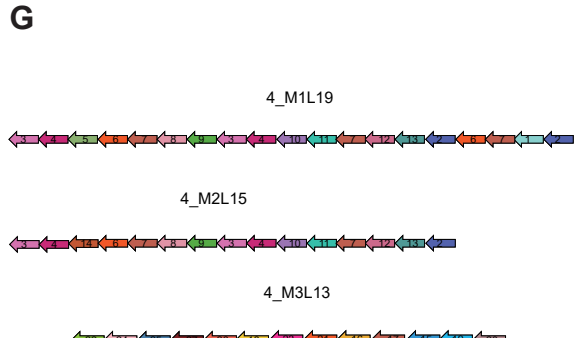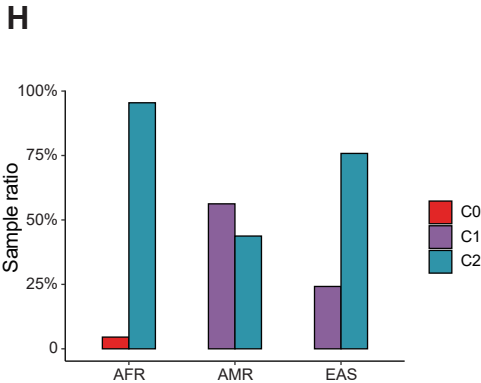

Supplement: qzae071_Supplementary_Data [file qzae071_supplementary_data.zip › Figure S10.pdf]

**A**

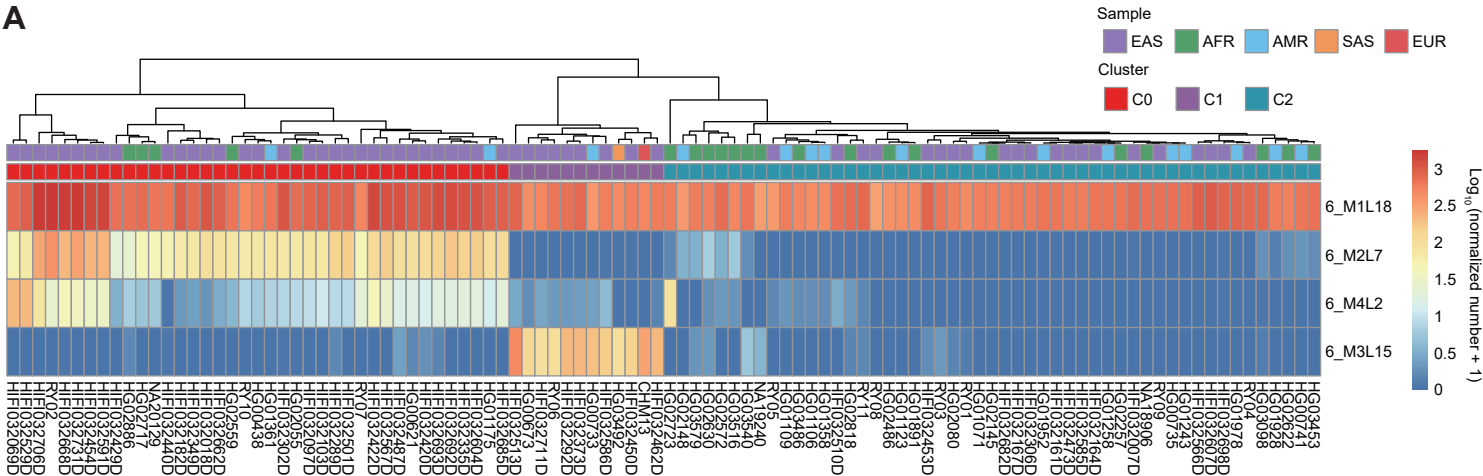

**B**

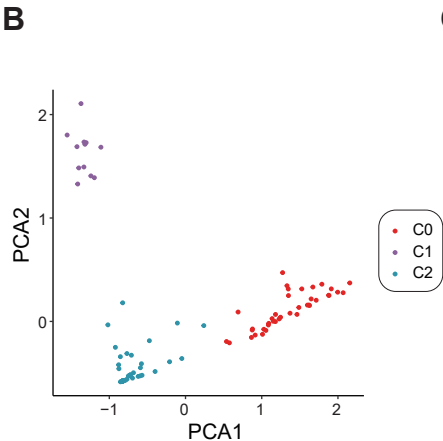

**C**

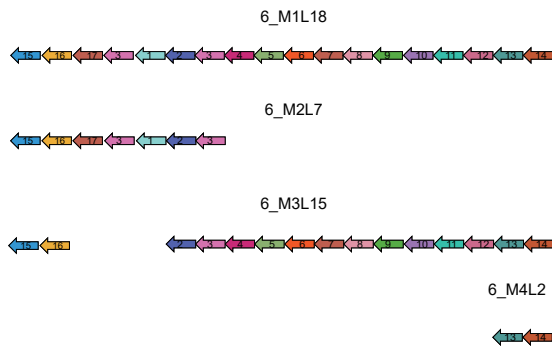

**D**

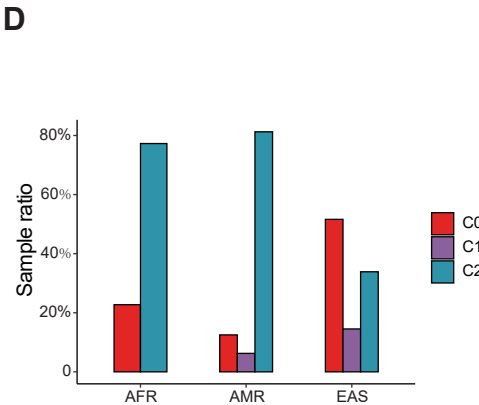

**E**

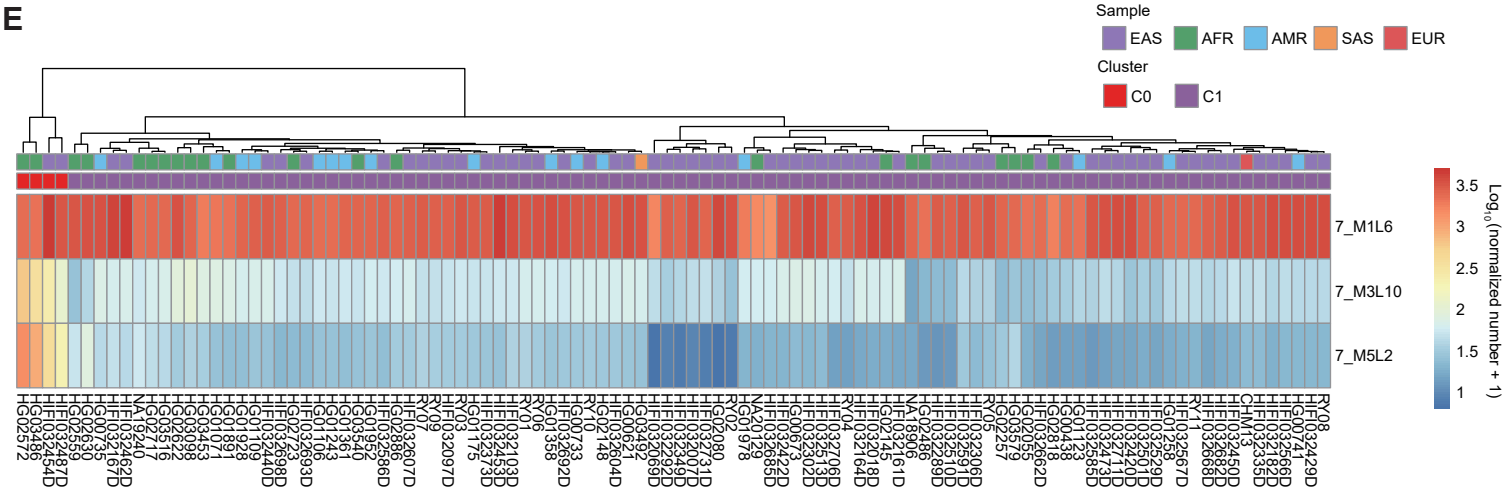

**F**

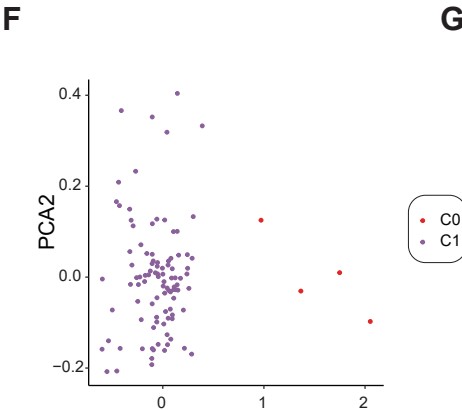

**G**

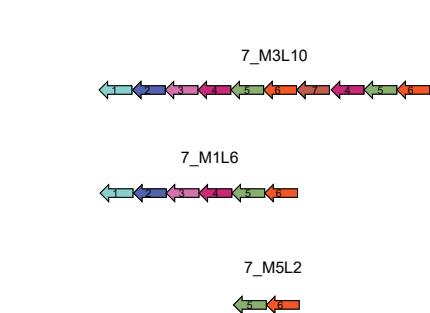

**H**

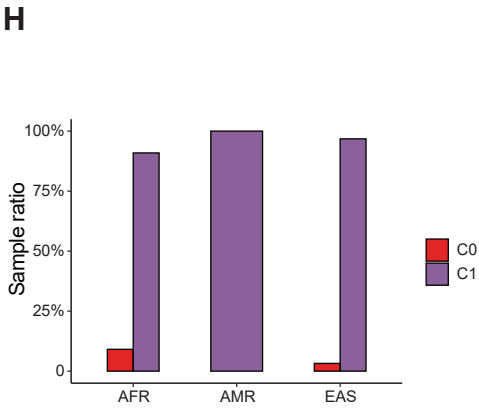

Supplement: qzae071_Supplementary_Data [file qzae071_supplementary_data.zip › Figure S11.pdf]

**A**

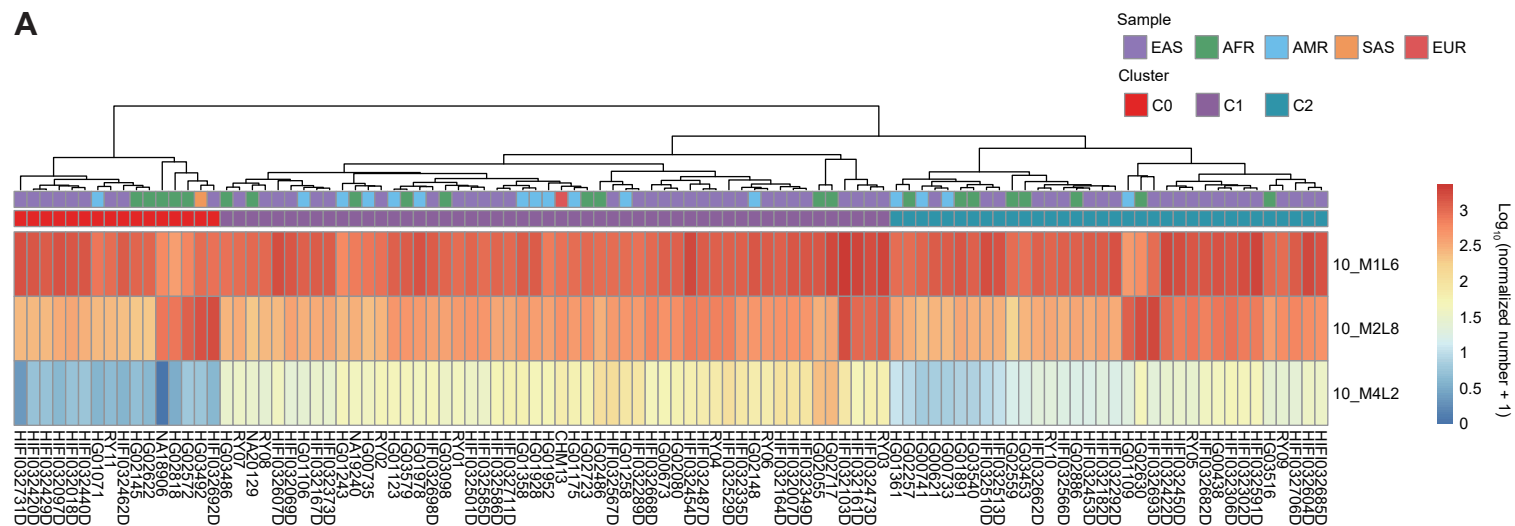

**B**

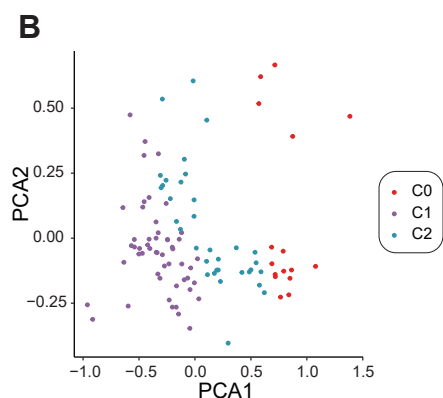

C

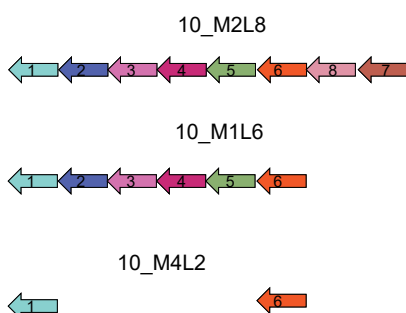

D

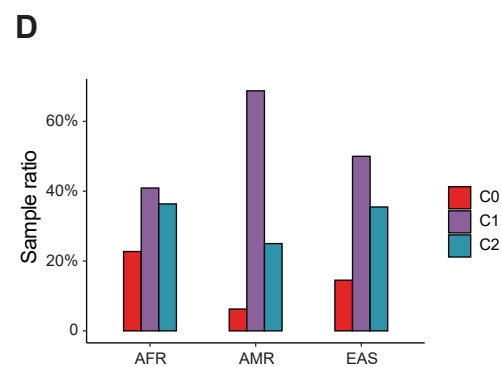

E

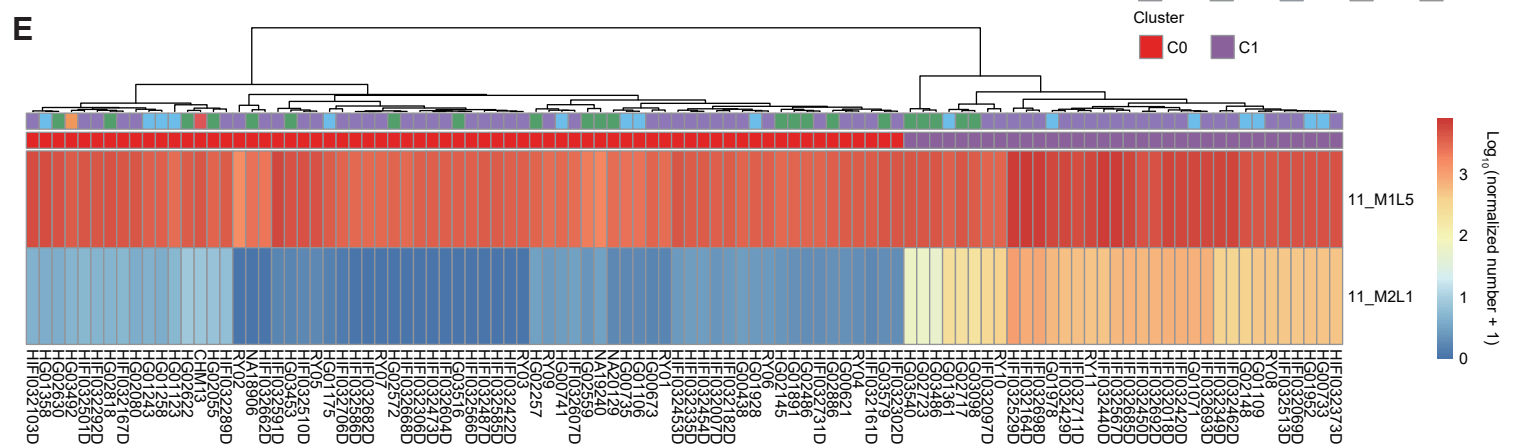**F**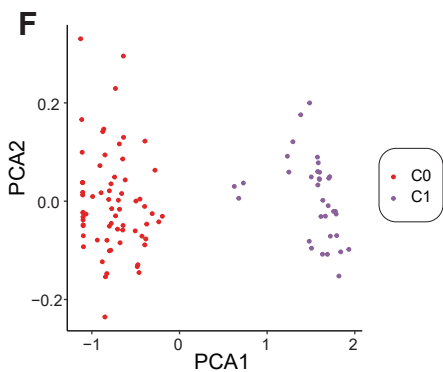

## G

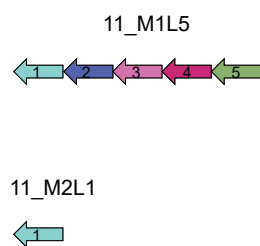

H

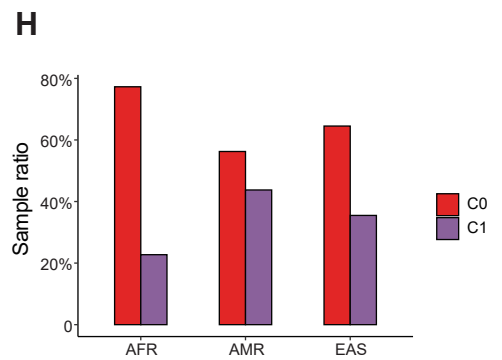

Supplement: qzae071_Supplementary_Data [file qzae071_supplementary_data.zip › Figure S12.pdf]

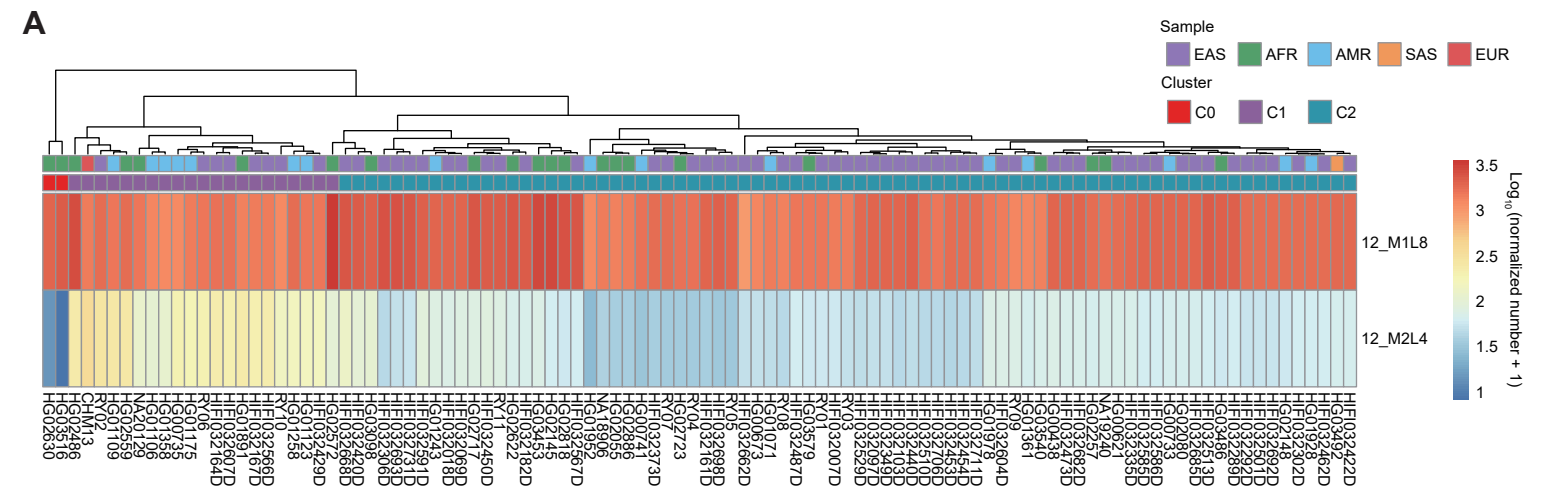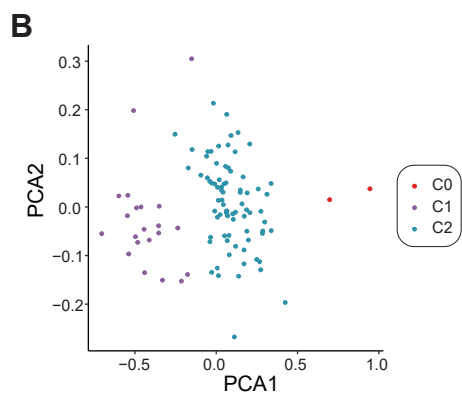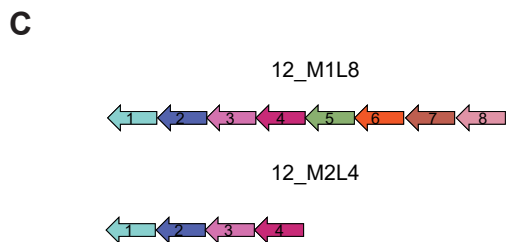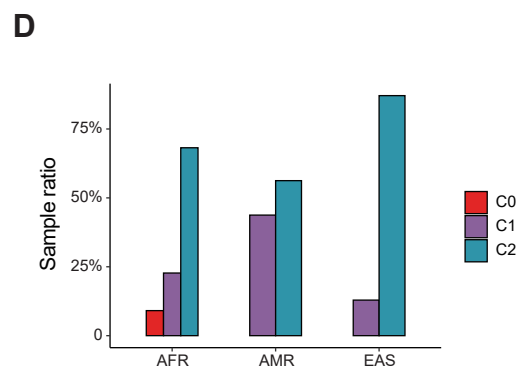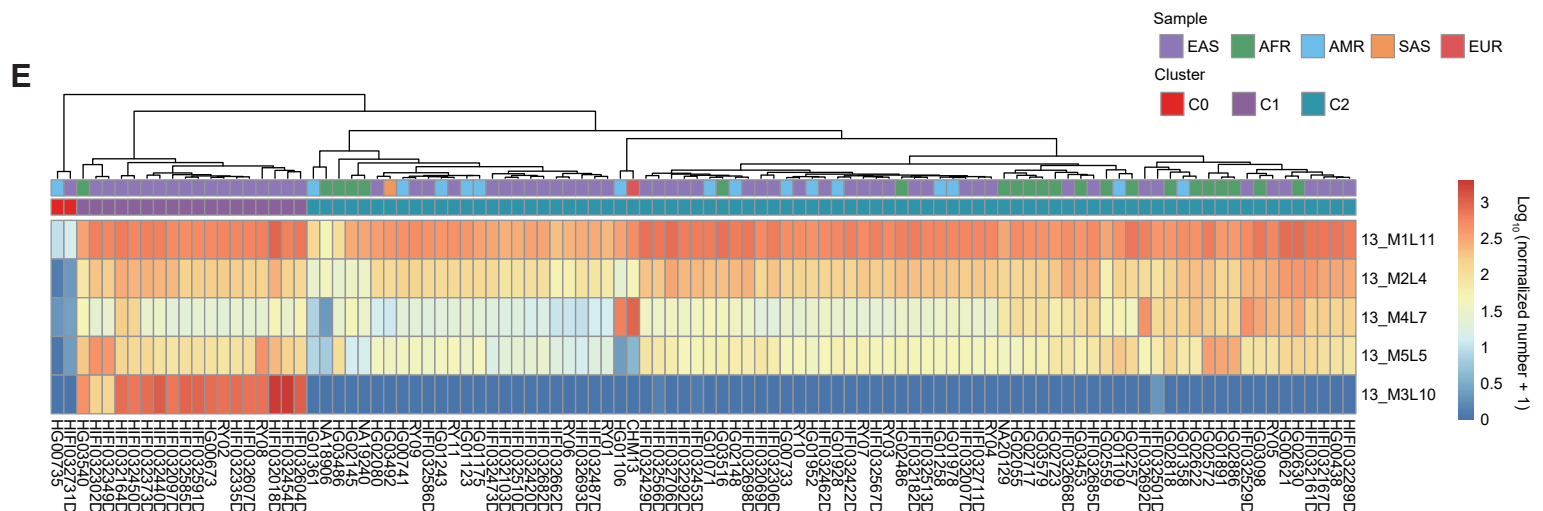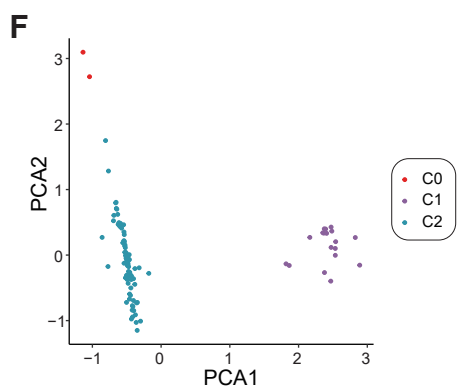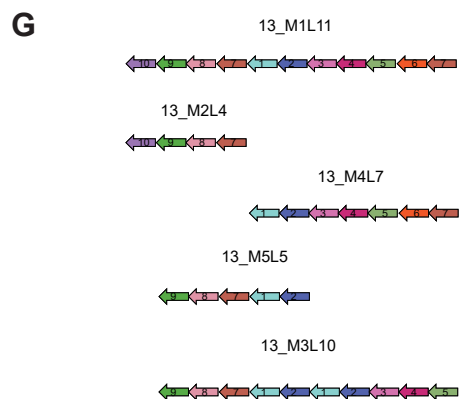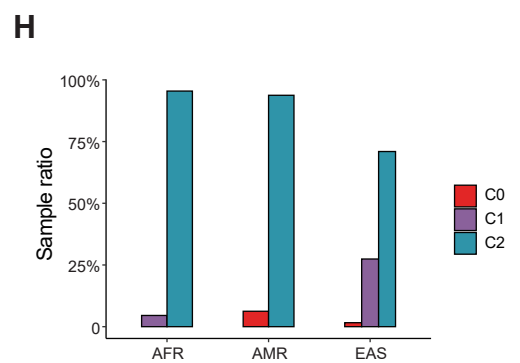

Supplement: qzae071_Supplementary_Data [file qzae071_supplementary_data.zip › Figure S13.pdf]

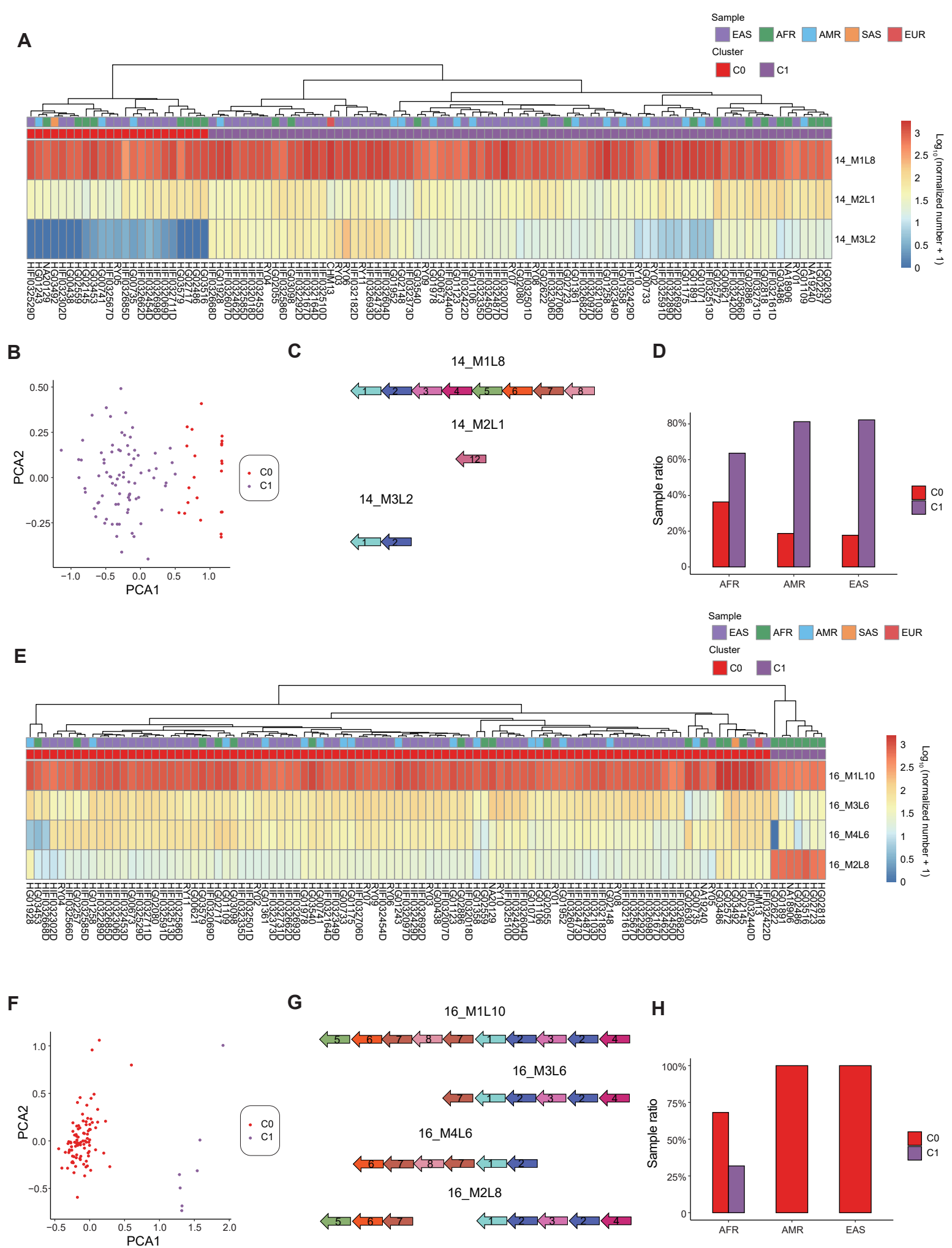

Supplement: qzae071_Supplementary_Data [file qzae071_supplementary_data.zip › Figure S14.pdf]

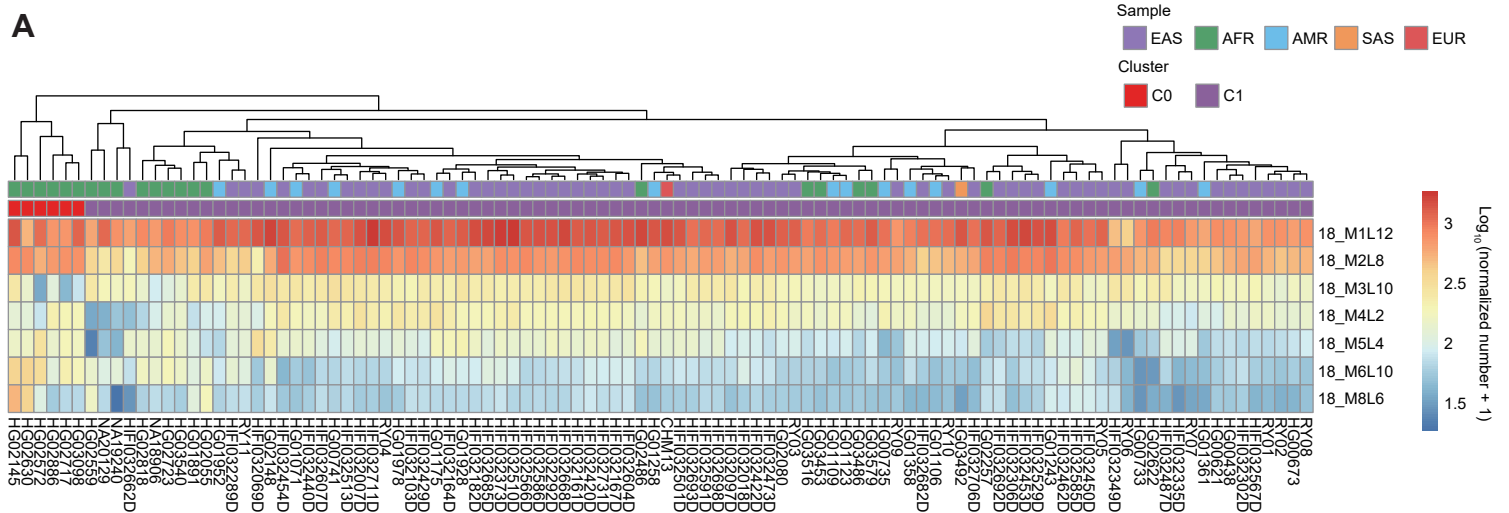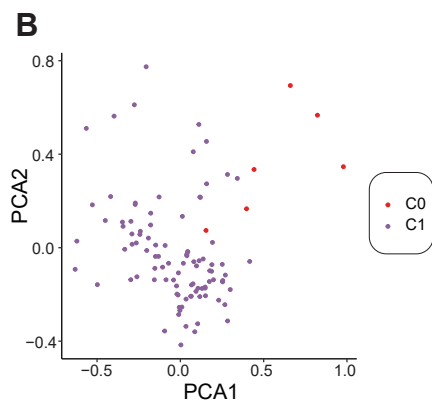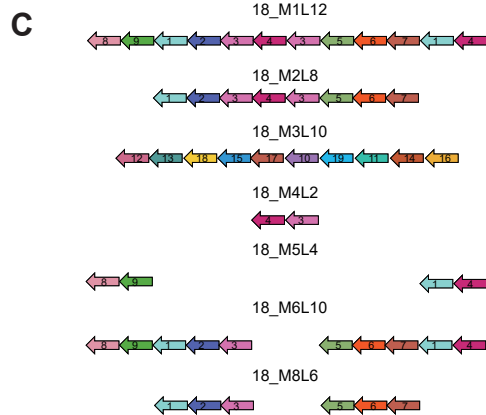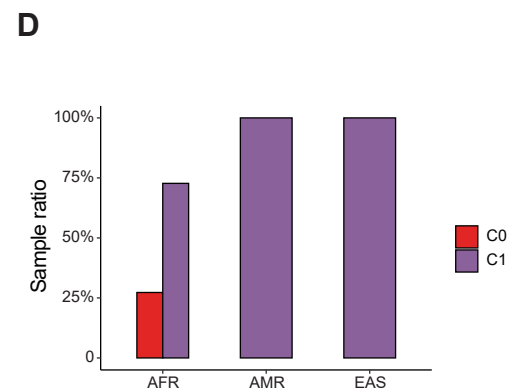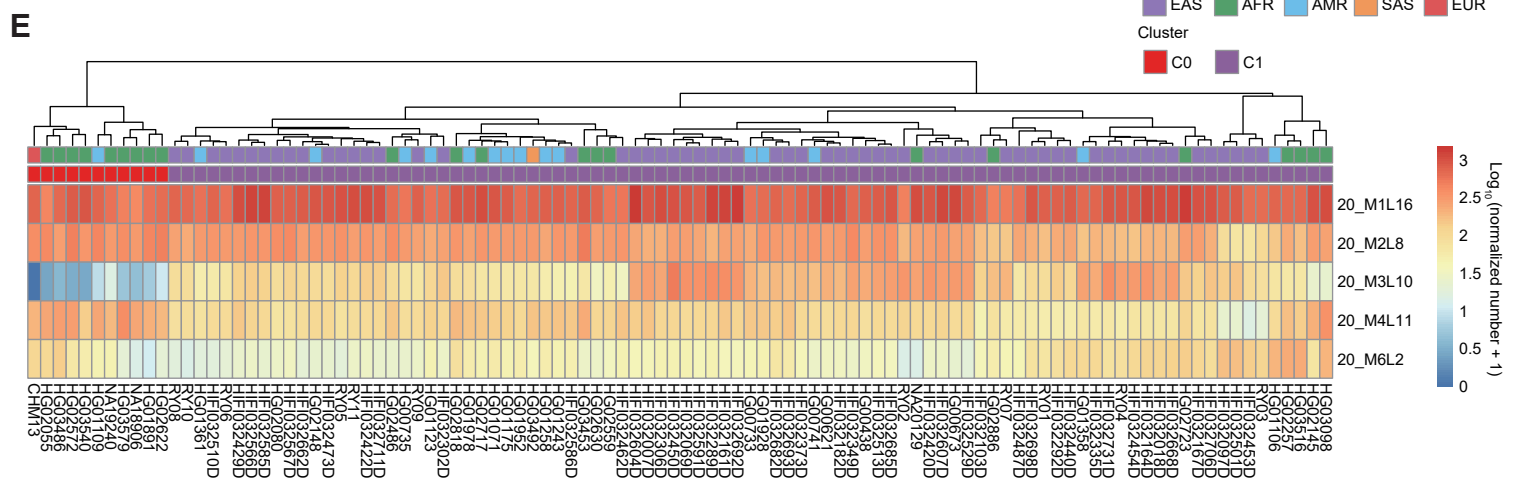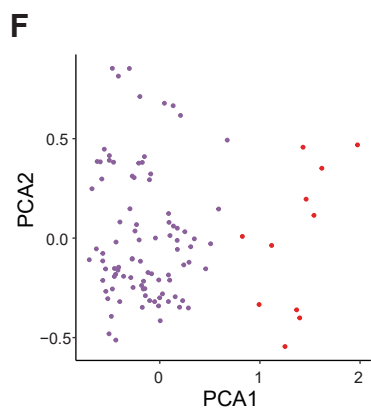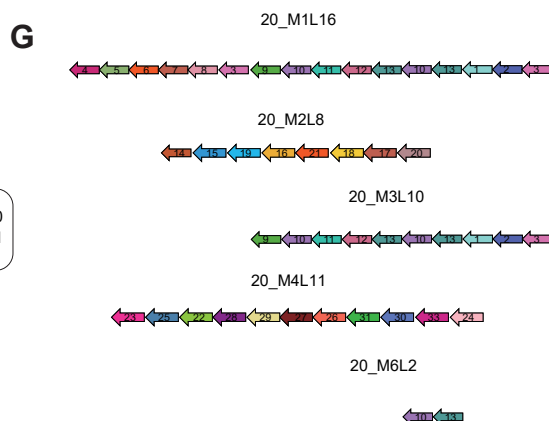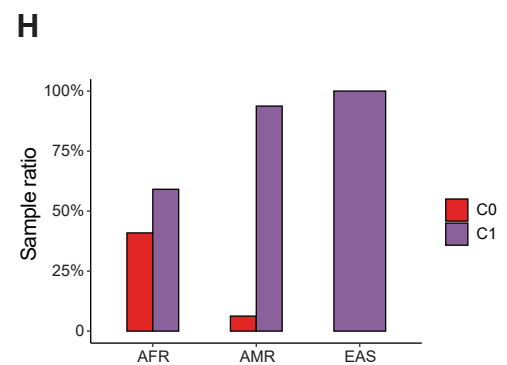

Supplement: qzae071_Supplementary_Data [file qzae071_supplementary_data.zip › Figure S15.pdf]

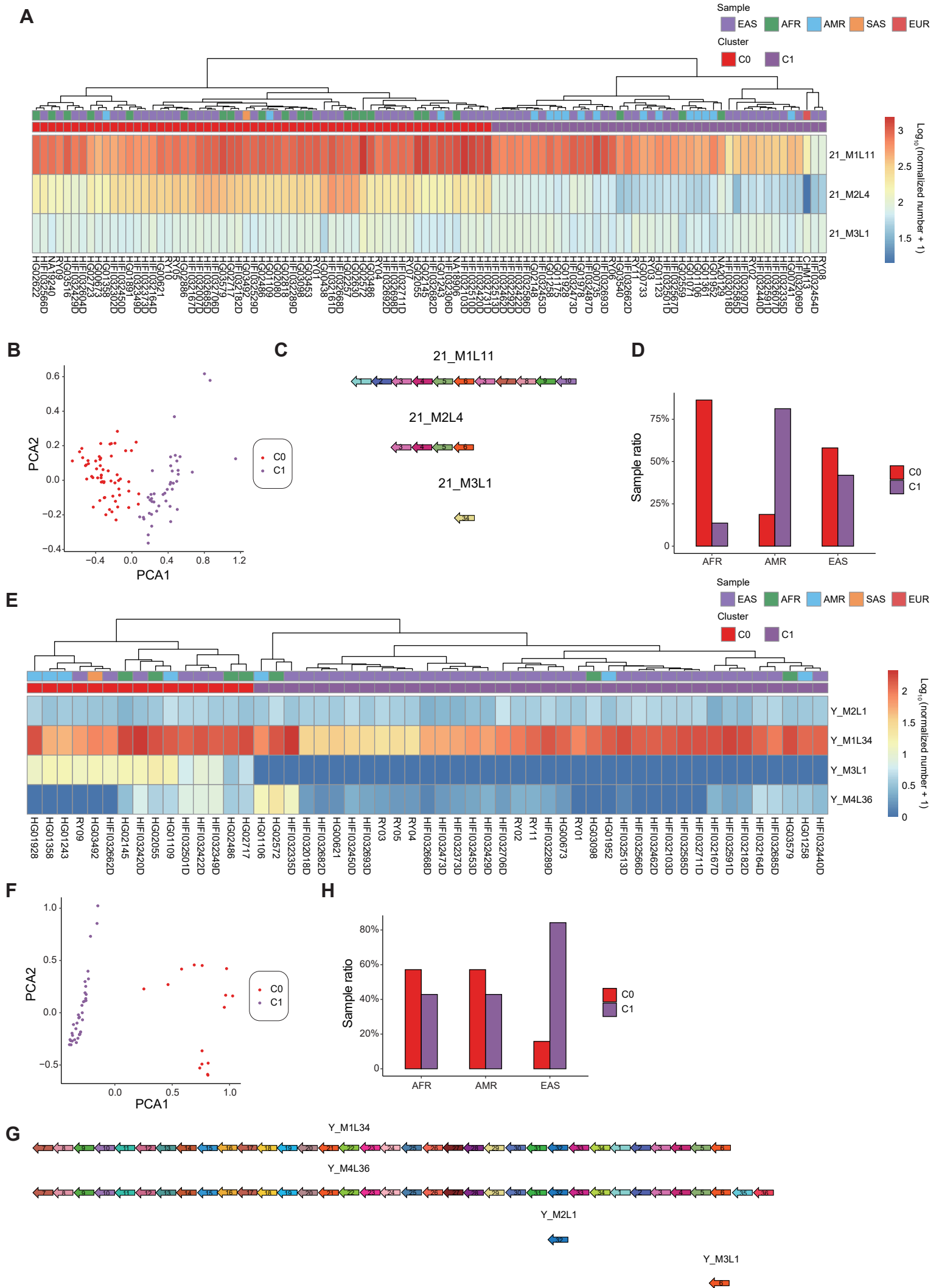

Supplement: qzae071_Supplementary_Data [file qzae071_supplementary_data.zip › Figure S16.pdf]

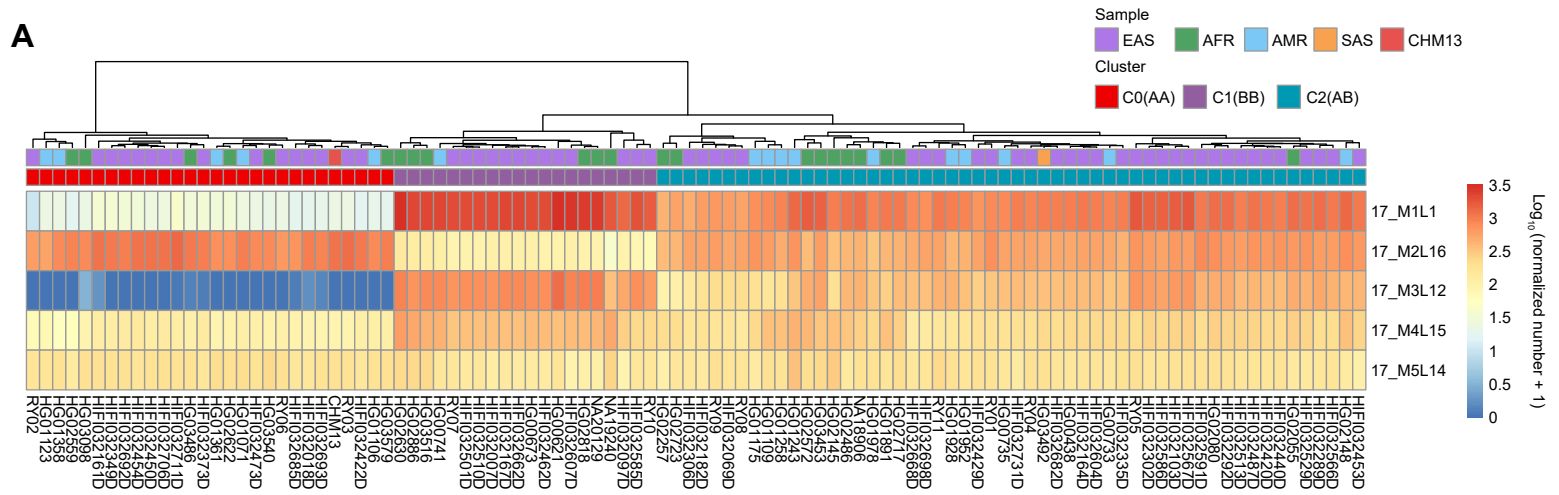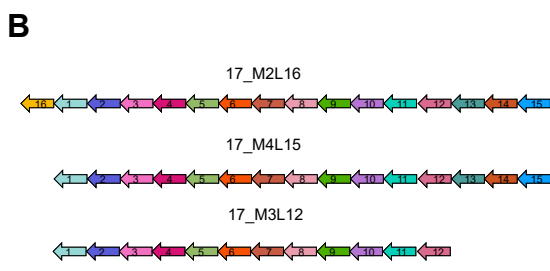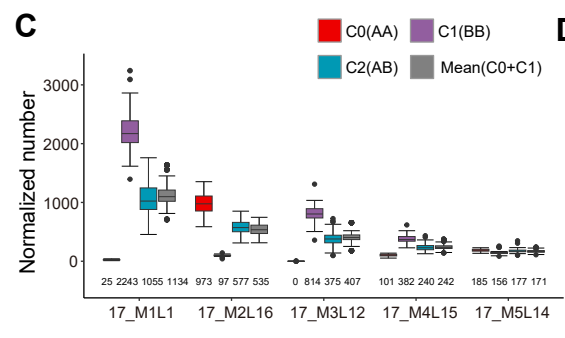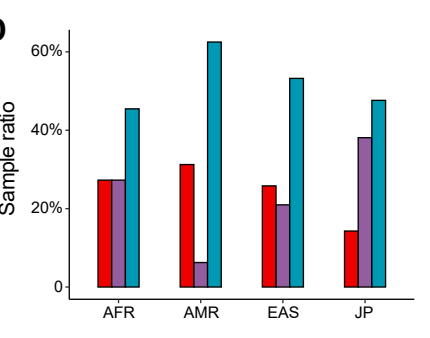

Supplement: qzae071_Supplementary_Data [file qzae071_supplementary_data.zip › Figure S17.pdf]

**A**

Chromosome 3

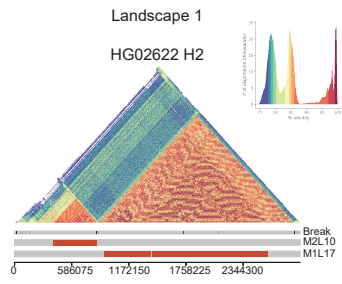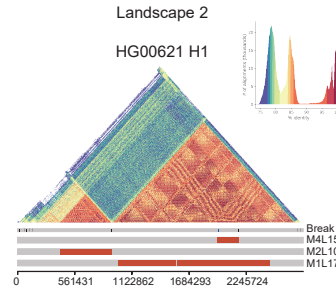**B**

Chromosome 4

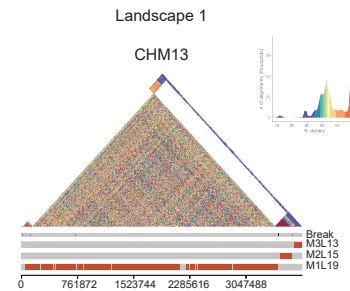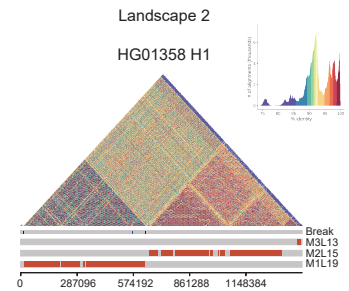**C**

Chromosome 6

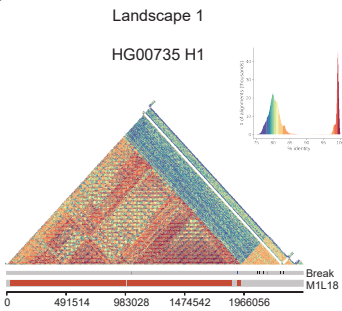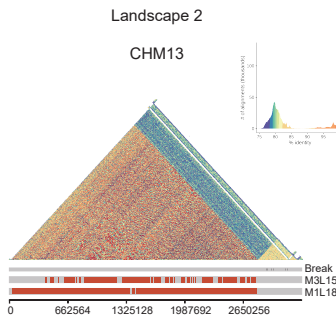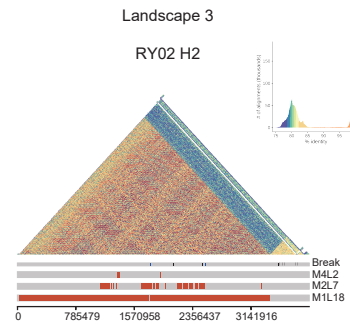

Chromosome 7

**D**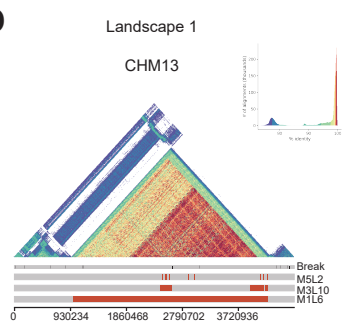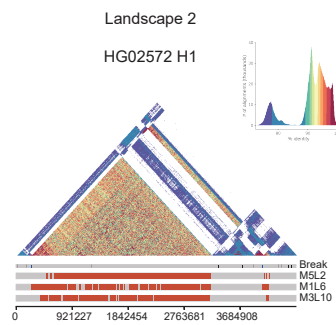

Chromosome 8

**E**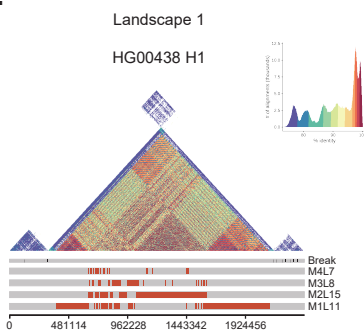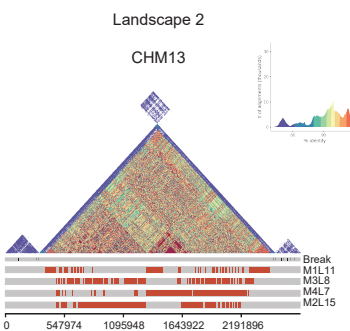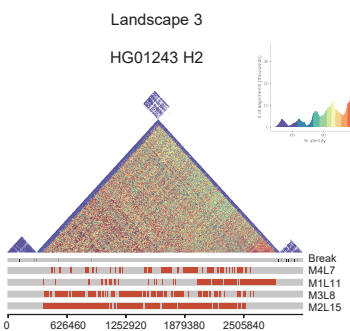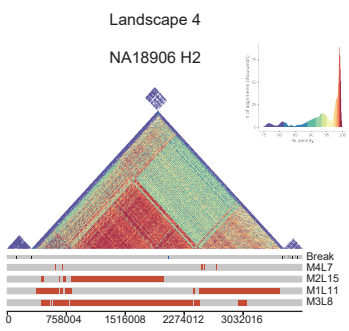**F**

Chromosome 12

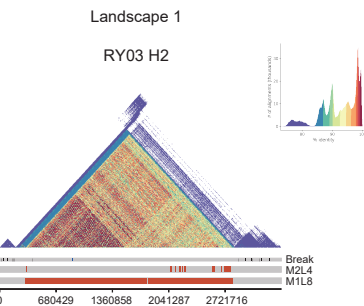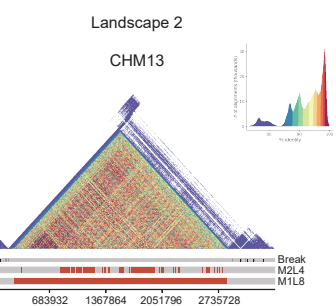

Supplement: qzae071_Supplementary_Data [file qzae071_supplementary_data.zip › Figure S18.pdf]

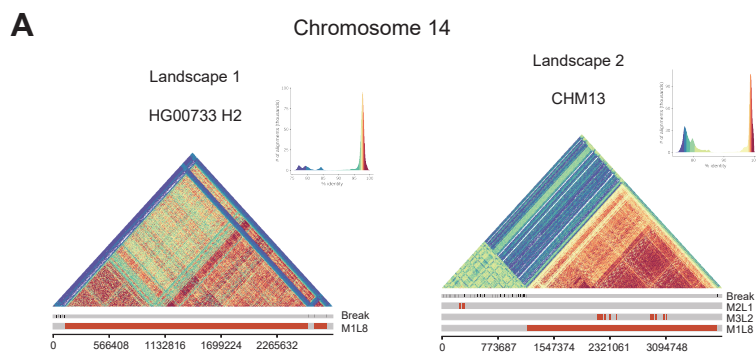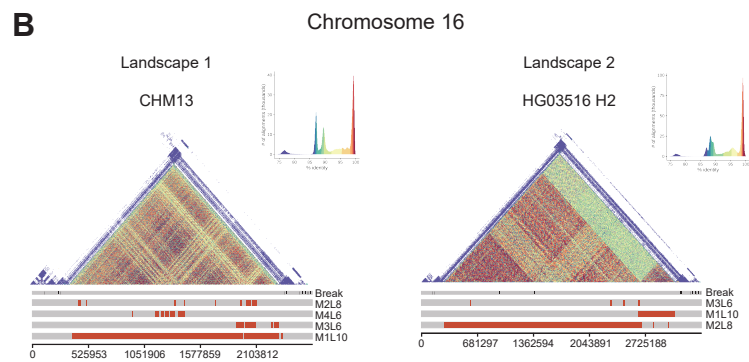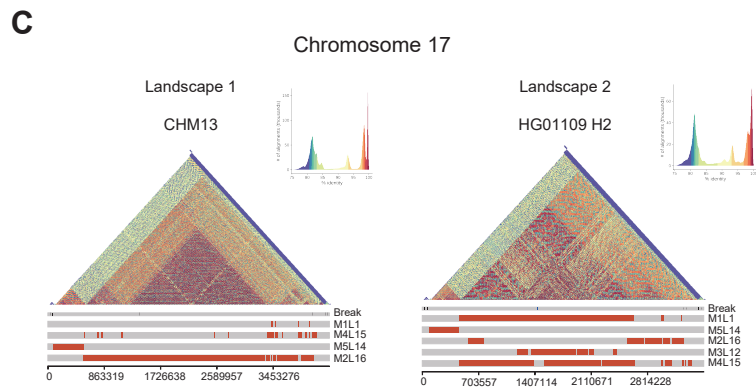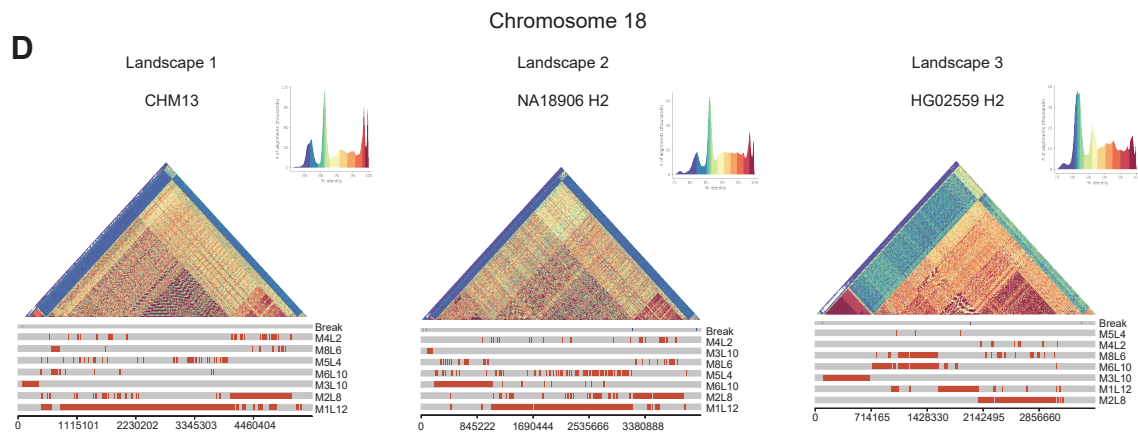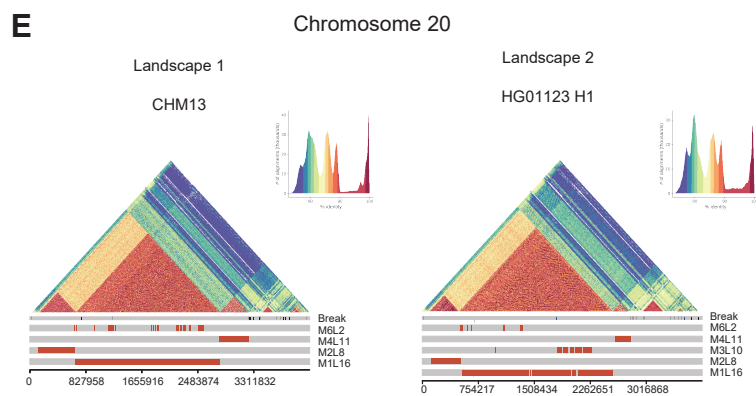

Supplement: qzae071_Supplementary_Data [file qzae071_supplementary_data.zip › Figure S19.pdf]

Chromosome 11 M1L5 clustering

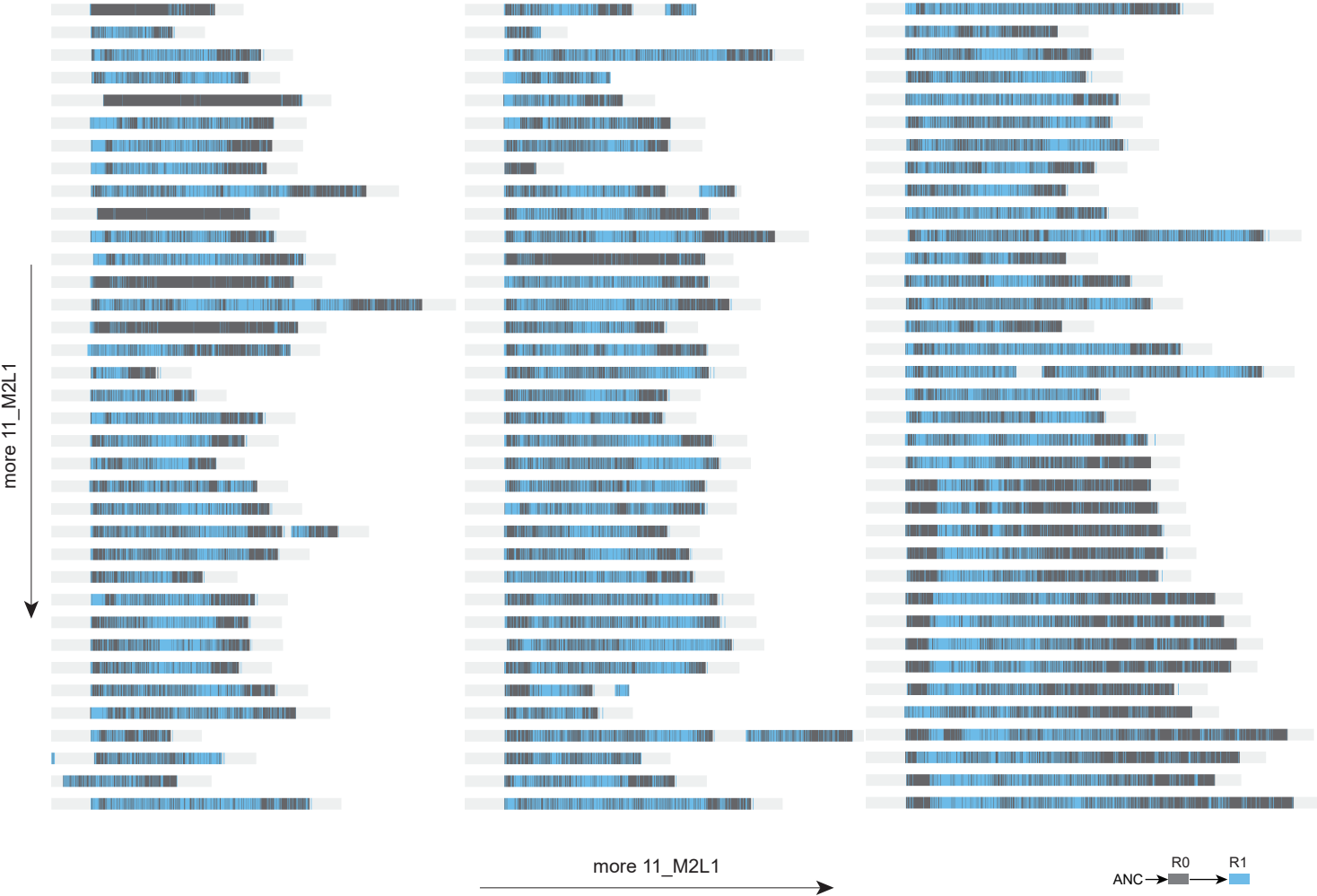

Supplement: qzae071_Supplementary_Data [file qzae071_supplementary_data.zip › Figure S20.pdf]

Chromosome 5 M2L8 clustering

Landscape 1

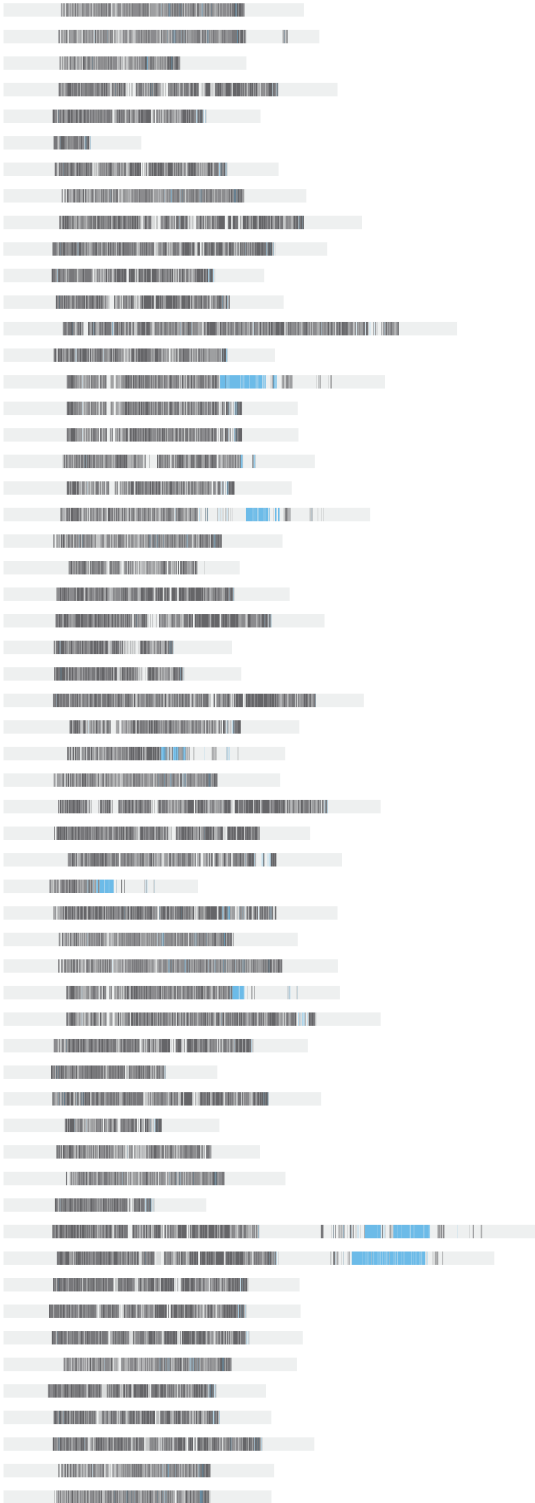

Landscape 2

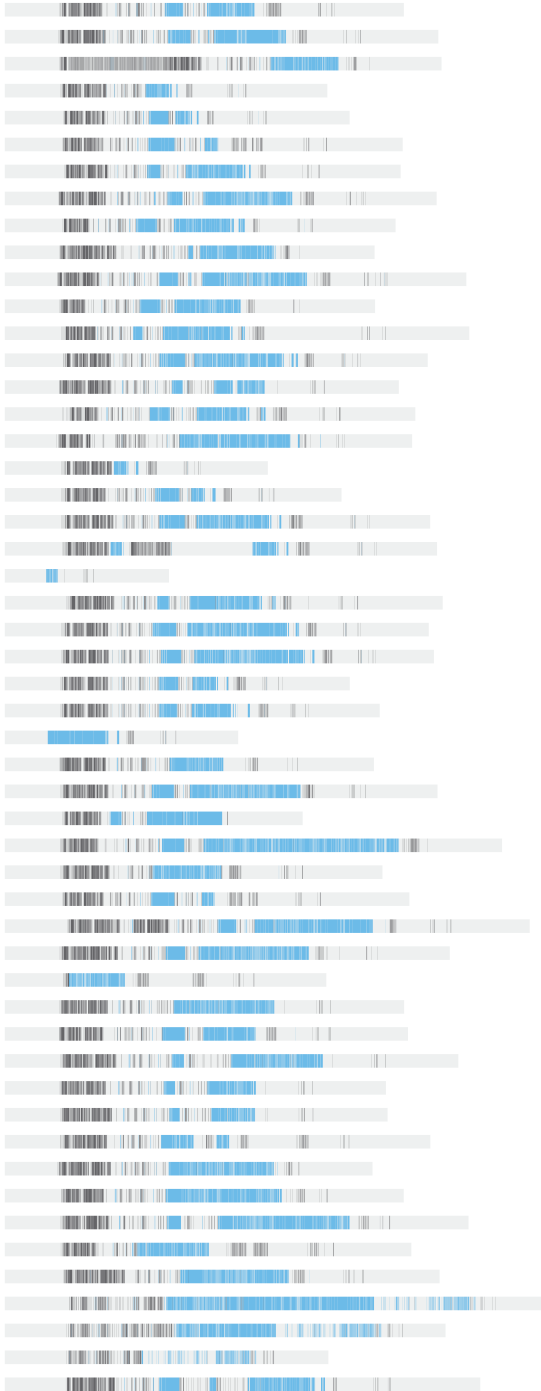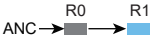

Supplement: qzae071_Supplementary_Data [file qzae071_supplementary_data.zip › Figure S22.pdf]

Chromosome 10 M1L6 clustering

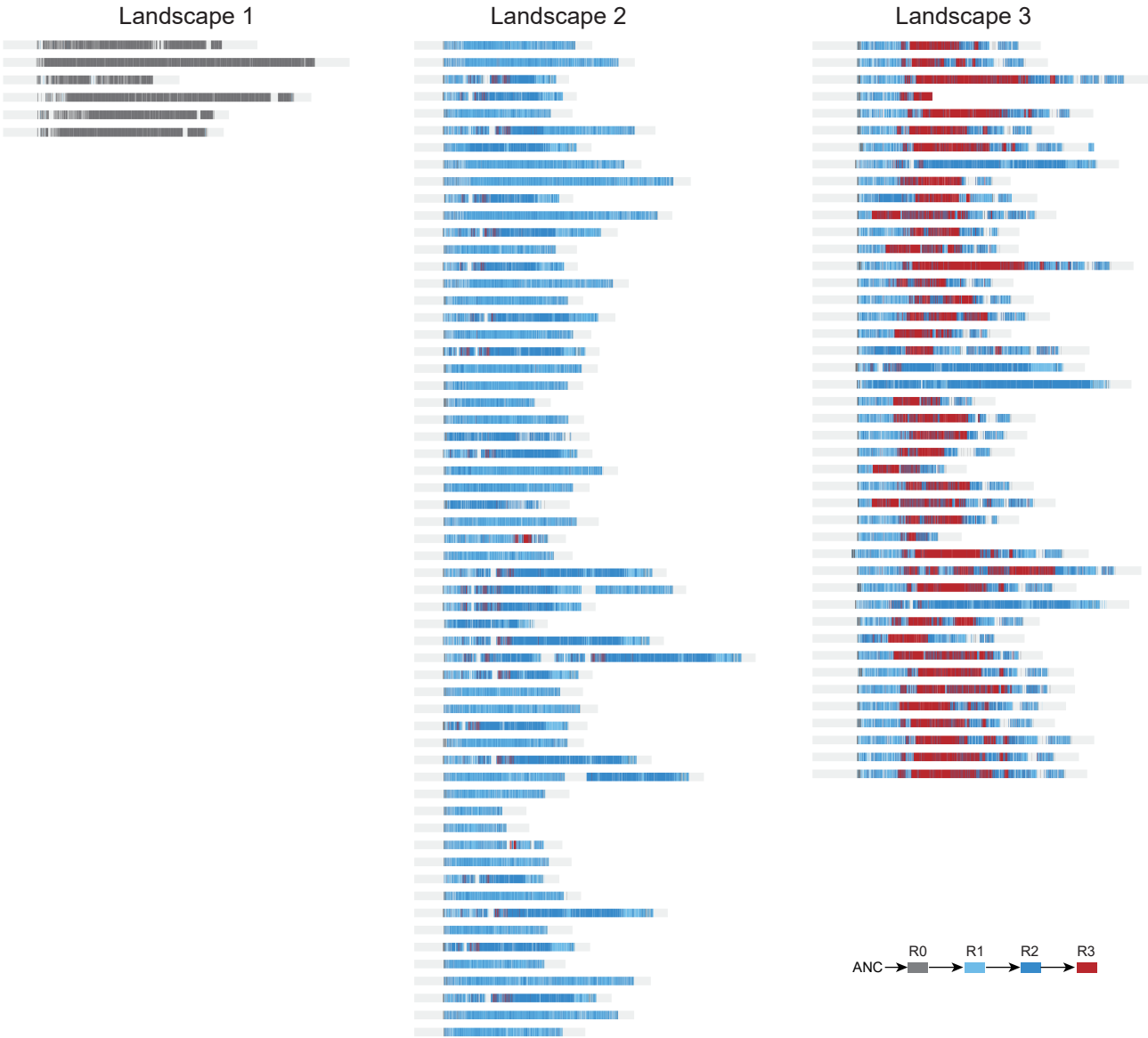

Supplement: qzae071_Supplementary_Data [file qzae071_supplementary_data.zip › Figure S23.pdf]

**A**

Chromosome 19

CHM13

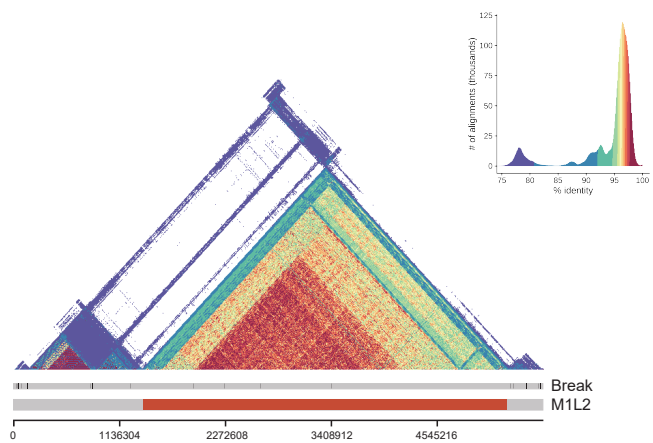**B**

Chromosome 22

CHM13

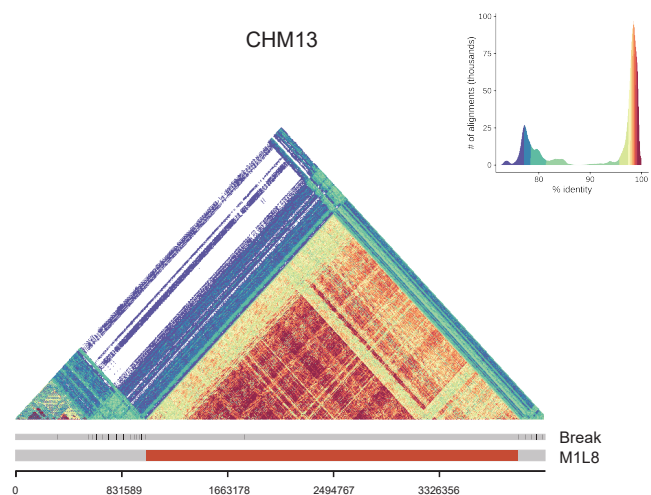**C**

Chromosome X

CHM13

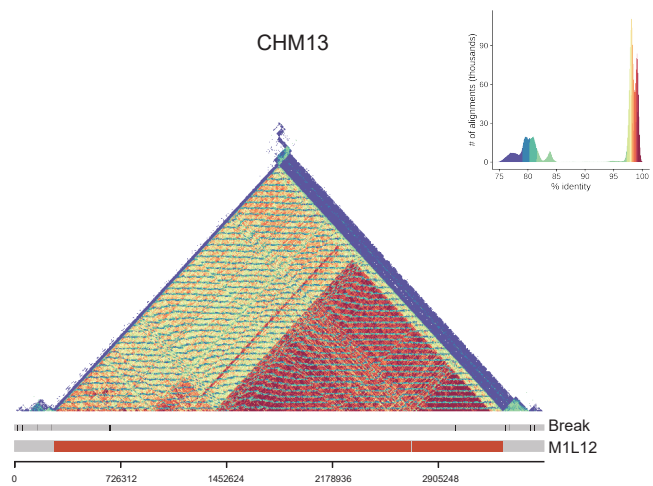

Supplement: qzae071_Supplementary_Data [file qzae071_supplementary_data.zip › Figure S24.pdf]

**A**

Chromosome 1

CHM13

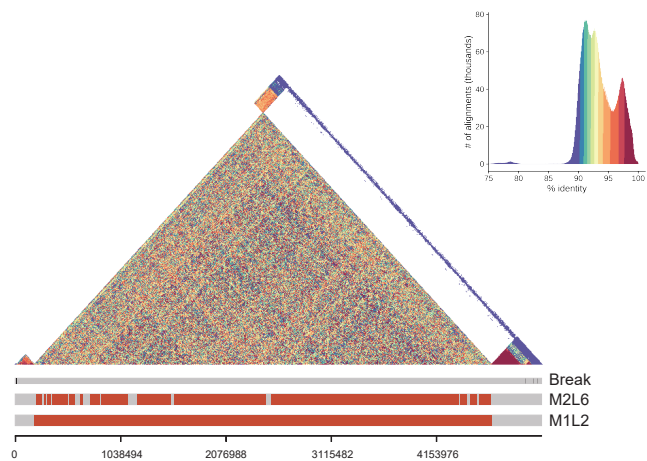**B**

Chromosome 2

CHM13

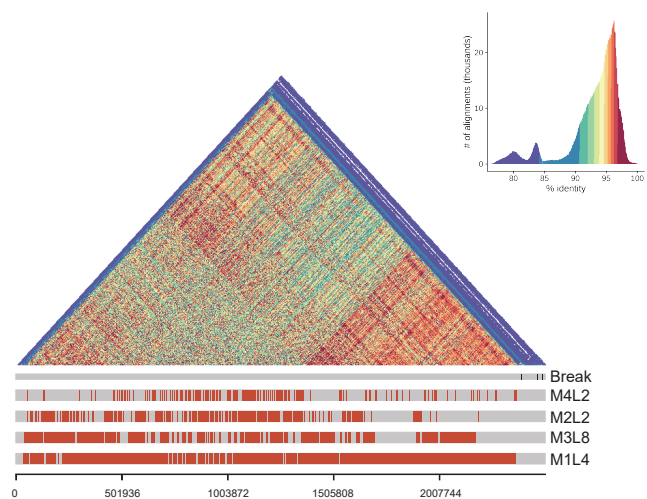**C**

Chromosome 9

CHM13

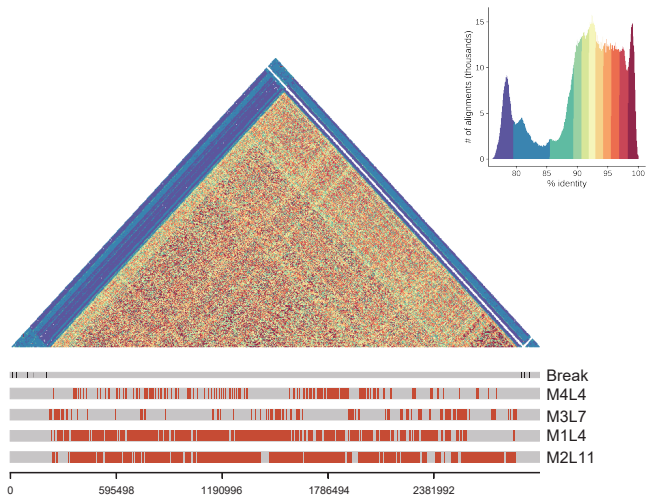**D**

Chromosome 15

CHM13

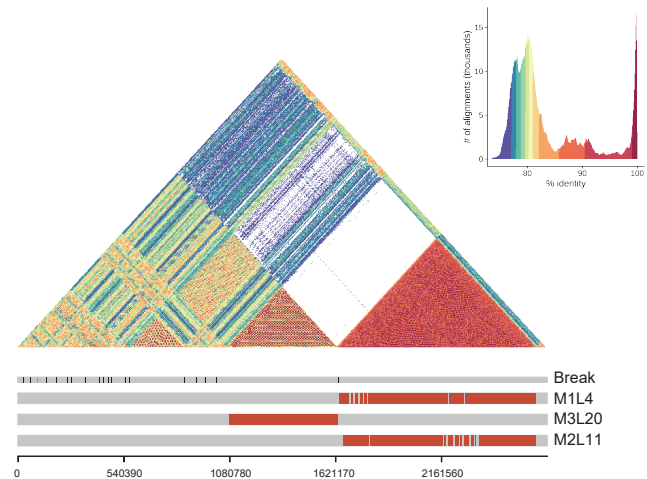

Supplement: qzae071_Supplementary_Data [file qzae071_supplementary_data.zip › Figure S25.pdf]

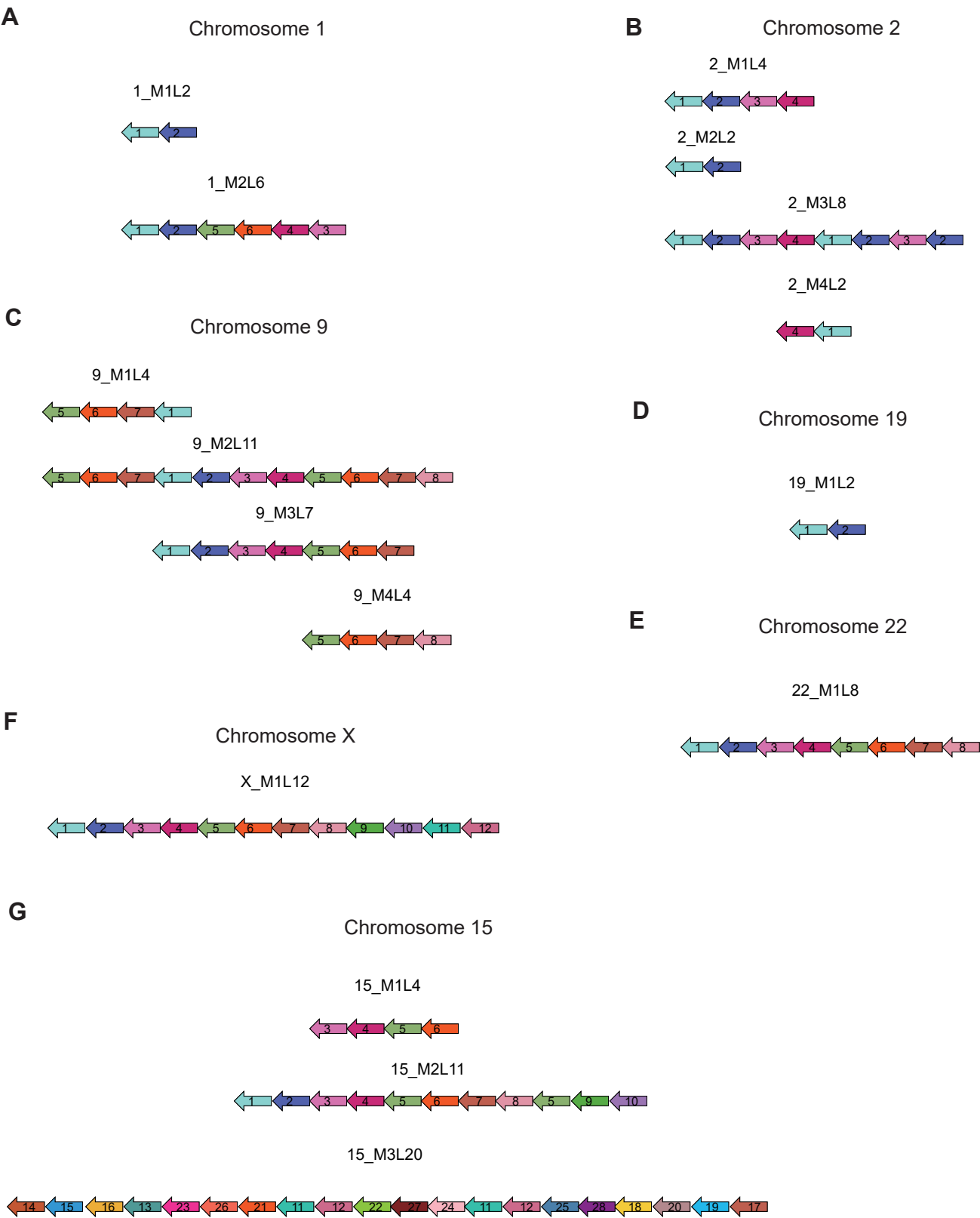

Supplement: qzae071_Supplementary_Data [file qzae071_supplementary_data.zip › Figure S26.pdf]

**A**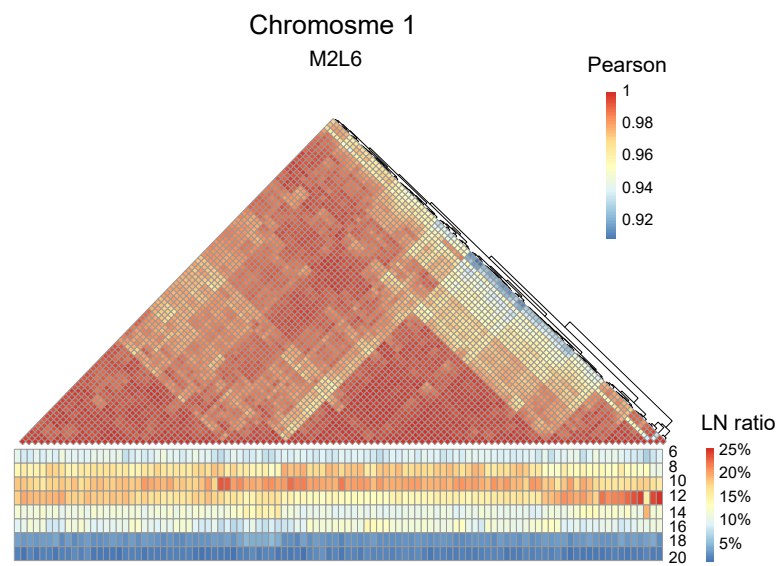**B**

HIFI032513D (1\_M2L6)

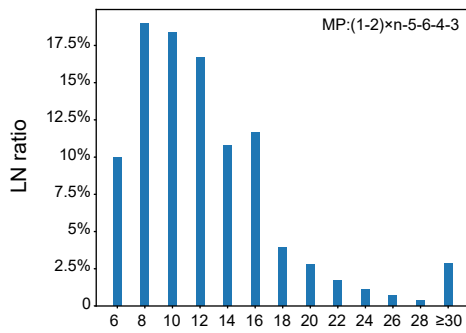**C**

RY07 (1\_M2L6)

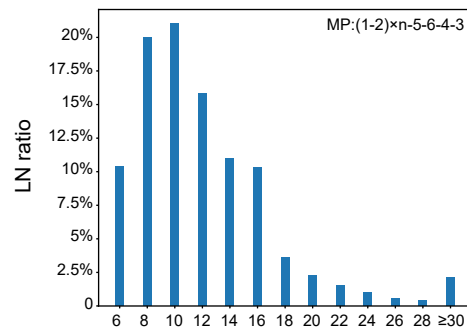**D**

HG01891 (1\_M2L6)

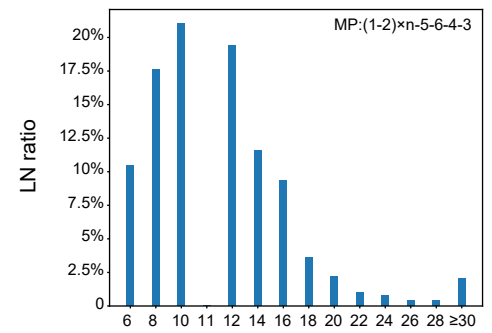**E**

CHM13 (1\_M2L6)

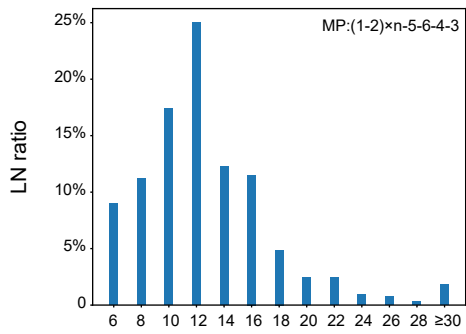**F**

HG01109 (1\_M2L6)

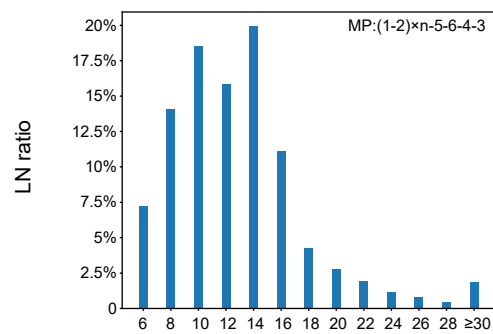**G**

1\_M2L6

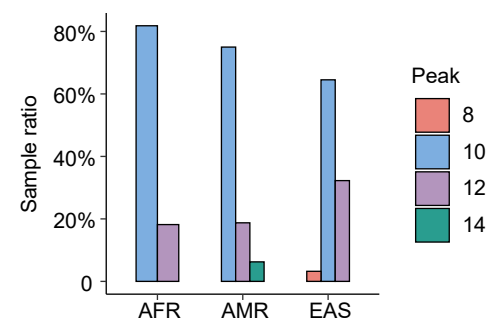

Supplement: qzae071_Supplementary_Data [file qzae071_supplementary_data.zip › Figure S27.pdf]

**A**

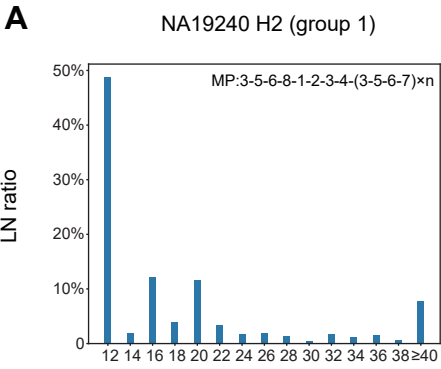

**B**

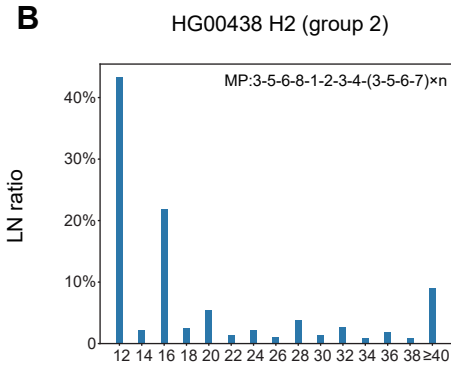

**C**

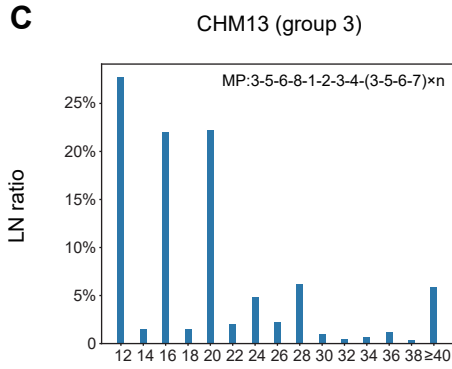

**D**

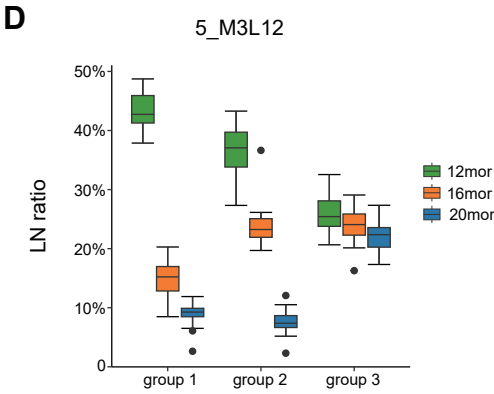

Supplement: qzae071_Supplementary_Data [file qzae071_supplementary_data.zip › Figure S28.pdf]

**A**

Chromosome 11 landscape 2  
(RY08 H1)

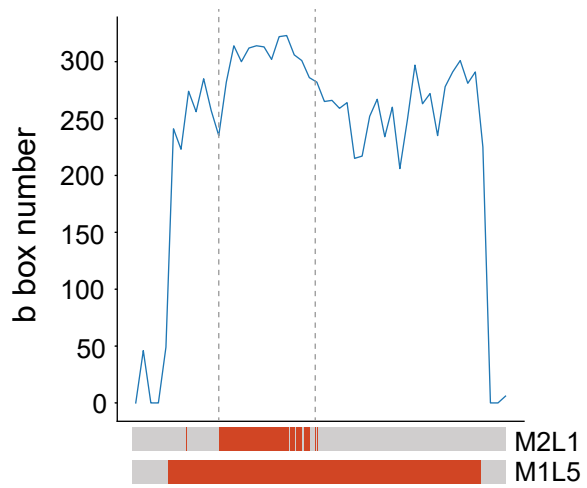**B**

Chromosome 10 landscape 3  
(RY07 H1)

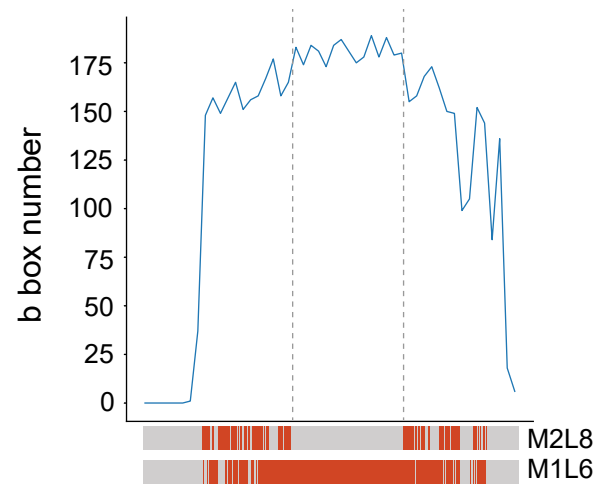

Supplement: qzae071_Supplementary_Data [file qzae071_supplementary_data.zip › Figure S29.pdf]

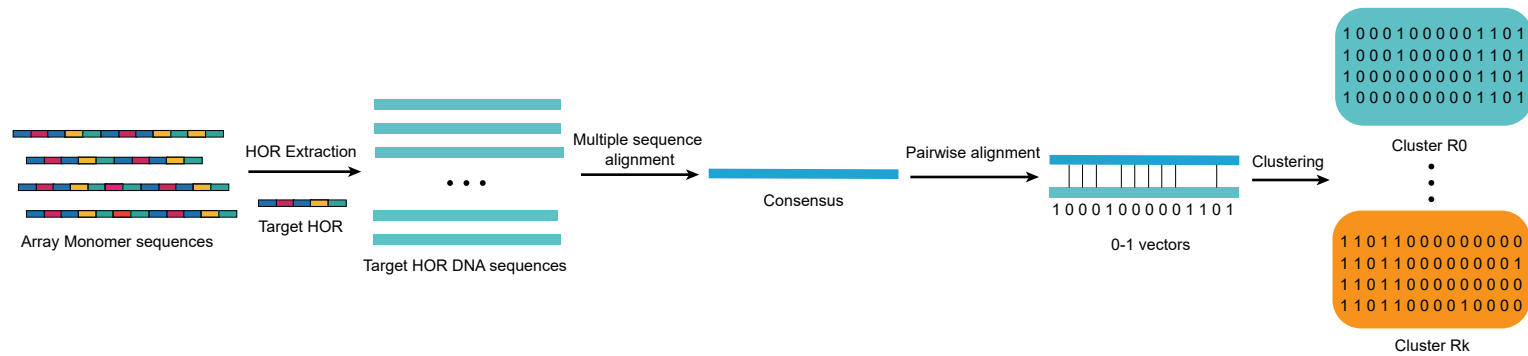

Supplement: qzae071_Supplementary_Data [file qzae071_supplementary_data.zip › Figure S30.pdf]

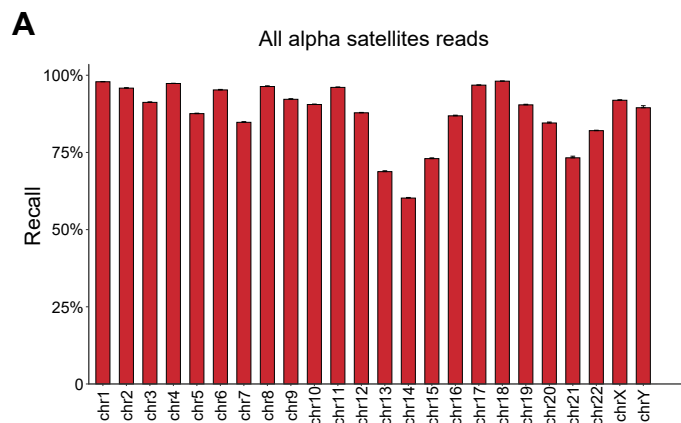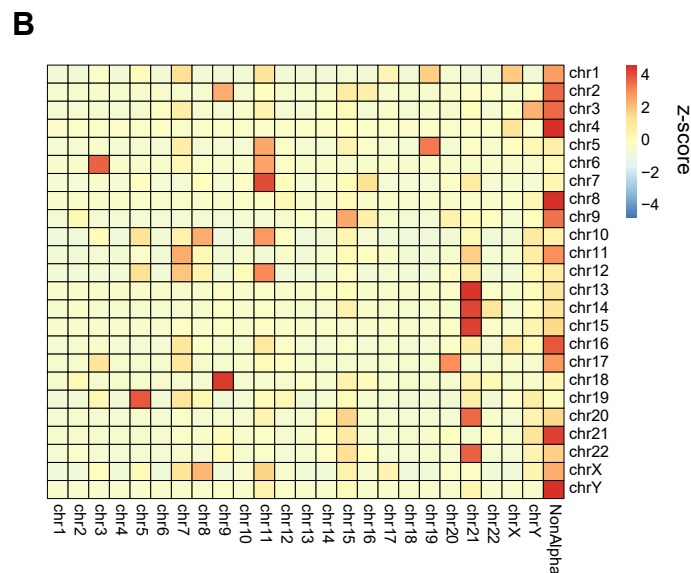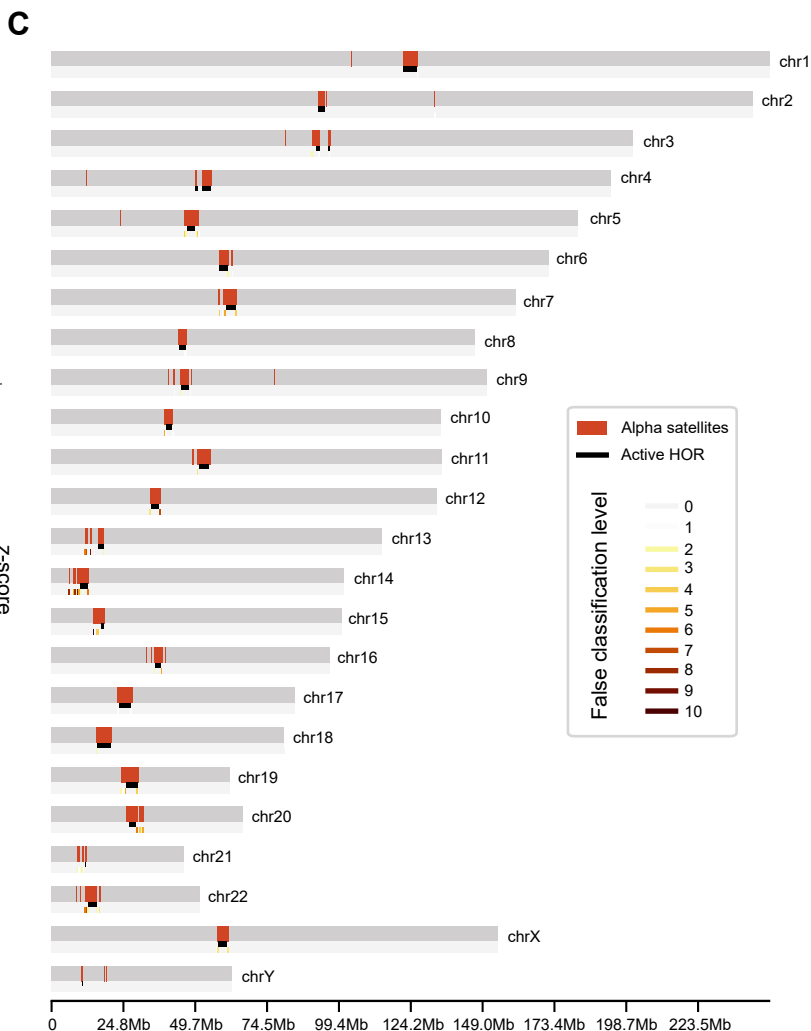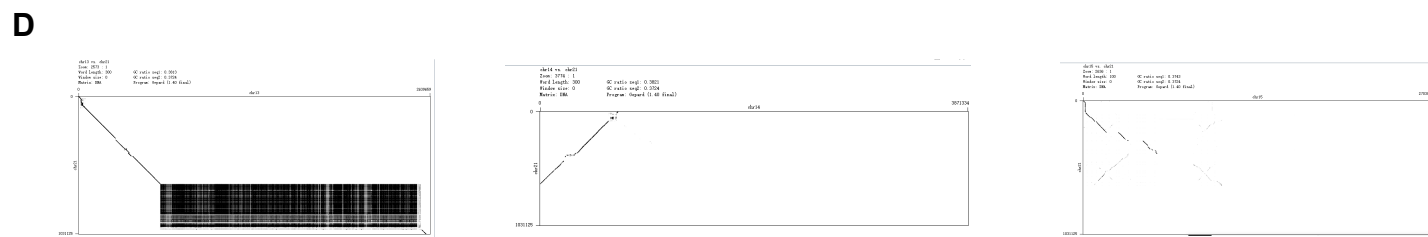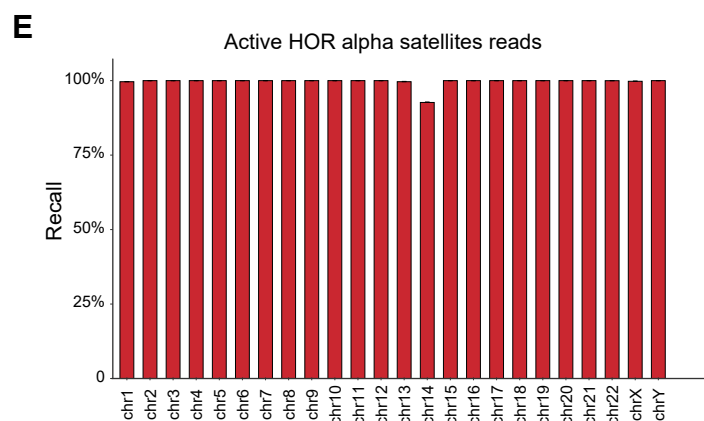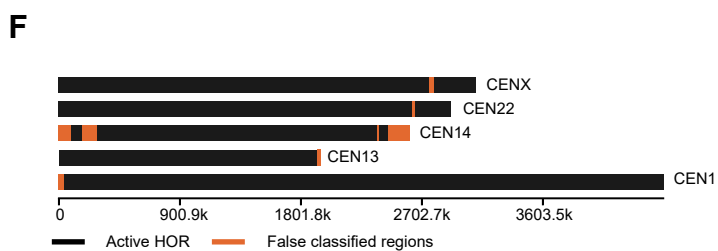

Supplement: qzae071_Supplementary_Data [file qzae071_supplementary_data.zip › Figure S1.pdf]

**A**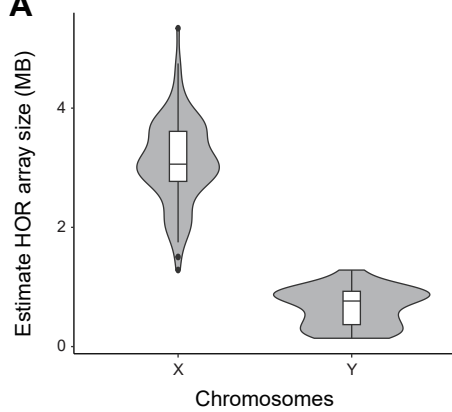**B**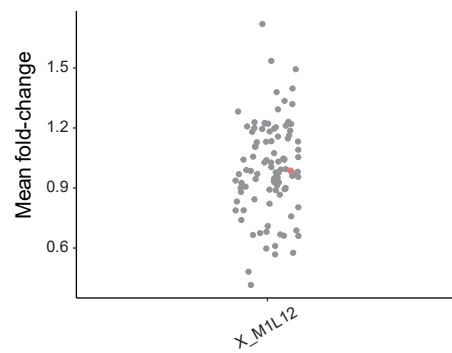**C**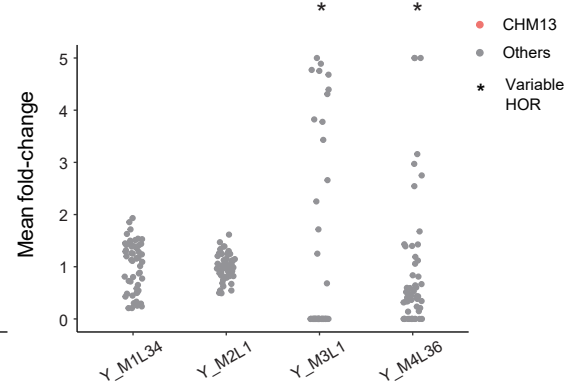

Supplement: qzae071_Supplementary_Data [file qzae071_supplementary_data.zip › Figure S2.pdf]

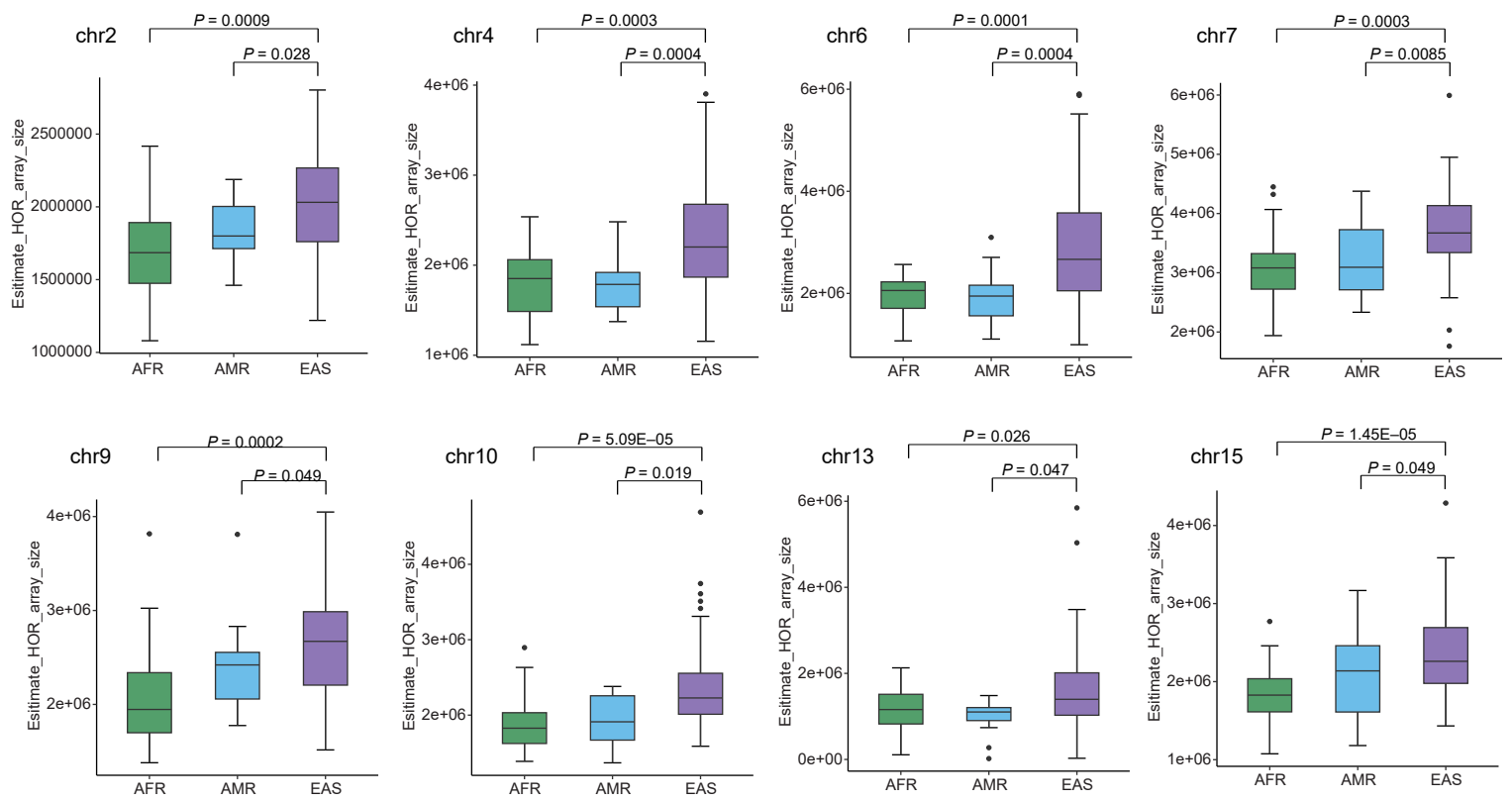

Supplement: qzae071_Supplementary_Data [file qzae071_supplementary_data.zip › Figure S3.pdf]

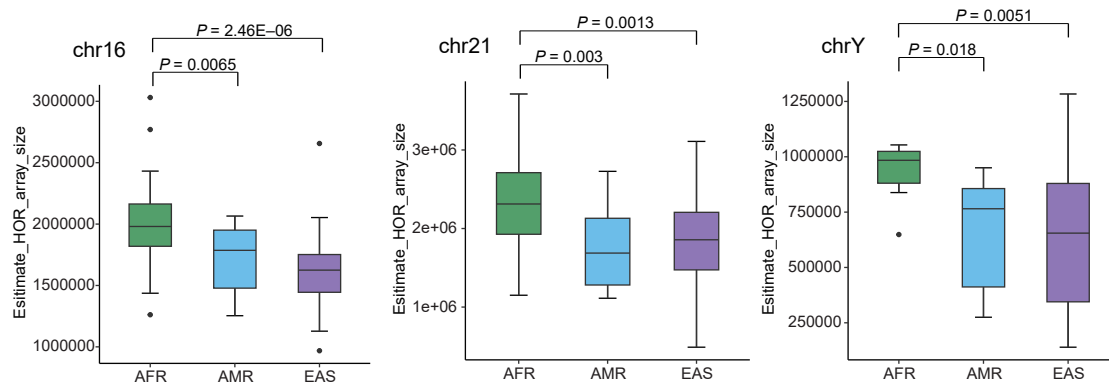

Supplement: qzae071_Supplementary_Data [file qzae071_supplementary_data.zip › Figure S4.pdf]
